# A multifunctional photothermal electrospun PLGA/MoS_2_@Pd nanofiber membrane for diabetic wound healing

**DOI:** 10.1093/rb/rbae143

**Published:** 2024-12-14

**Authors:** Zhengrong Chen, Quansheng Mo, Dandan Mo, Xiaomin Pei, Anru Liang, Jinhong Cai, Bo Zhou, Li Zheng, Hongmian Li, Feiying Yin, Jinmin Zhao

**Affiliations:** Guangxi Engineering Center in Biomedical Material for Tissue and Organ Regeneration, Collaborative Innovation Centre of Regenerative Medicine and Medical BioResource Development and Application Co-constructed By the Province and Ministry, Guangxi Key Laboratory of Regenerative Medicine, The First Affiliated Hospital of Guangxi Medical University, Nanning, Guangxi 530021, China; National & Regional United Engineering Lab of Tissue Engineering, Department of Orthopedics, Southwest Hospital, Third Military Medical University (Army Medical University), Chongqing 400038, China; Department of Traditional Chinese Medicine, The Ninth People's Hospital of Nanning, Binyang, Guangxi 530409, China; Guangxi Engineering Center in Biomedical Material for Tissue and Organ Regeneration, Collaborative Innovation Centre of Regenerative Medicine and Medical BioResource Development and Application Co-constructed By the Province and Ministry, Guangxi Key Laboratory of Regenerative Medicine, The First Affiliated Hospital of Guangxi Medical University, Nanning, Guangxi 530021, China; Guangxi Engineering Center in Biomedical Material for Tissue and Organ Regeneration, Collaborative Innovation Centre of Regenerative Medicine and Medical BioResource Development and Application Co-constructed By the Province and Ministry, Guangxi Key Laboratory of Regenerative Medicine, The First Affiliated Hospital of Guangxi Medical University, Nanning, Guangxi 530021, China; Life Sciences Institute, Guangxi Medical University, Nanning, Guangxi 530021, China; Department of Burns and Plastic Surgery, The Third Affiliated Hospital of Guangxi Medical University & The Second Nanning People's Hospital, Nanning 530031, China; Guangxi Engineering Center in Biomedical Material for Tissue and Organ Regeneration, Collaborative Innovation Centre of Regenerative Medicine and Medical BioResource Development and Application Co-constructed By the Province and Ministry, Guangxi Key Laboratory of Regenerative Medicine, The First Affiliated Hospital of Guangxi Medical University, Nanning, Guangxi 530021, China; Guangxi Engineering Center in Biomedical Material for Tissue and Organ Regeneration, Collaborative Innovation Centre of Regenerative Medicine and Medical BioResource Development and Application Co-constructed By the Province and Ministry, Guangxi Key Laboratory of Regenerative Medicine, The First Affiliated Hospital of Guangxi Medical University, Nanning, Guangxi 530021, China; Guangxi Engineering Center in Biomedical Material for Tissue and Organ Regeneration, Collaborative Innovation Centre of Regenerative Medicine and Medical BioResource Development and Application Co-constructed By the Province and Ministry, Guangxi Key Laboratory of Regenerative Medicine, The First Affiliated Hospital of Guangxi Medical University, Nanning, Guangxi 530021, China; Life Sciences Institute, Guangxi Medical University, Nanning, Guangxi 530021, China; Department of Plastic and Reconstructive Surgery, The People’s Hospital of Guangxi Zhuang Autonomous Region & Research Center of Medical Sciences, Guangxi Academy of Medical Sciences, Nanning 530021, China; Guangxi Engineering Center in Biomedical Material for Tissue and Organ Regeneration, Collaborative Innovation Centre of Regenerative Medicine and Medical BioResource Development and Application Co-constructed By the Province and Ministry, Guangxi Key Laboratory of Regenerative Medicine, The First Affiliated Hospital of Guangxi Medical University, Nanning, Guangxi 530021, China; Life Sciences Institute, Guangxi Medical University, Nanning, Guangxi 530021, China; Guangxi Engineering Center in Biomedical Material for Tissue and Organ Regeneration, Collaborative Innovation Centre of Regenerative Medicine and Medical BioResource Development and Application Co-constructed By the Province and Ministry, Guangxi Key Laboratory of Regenerative Medicine, The First Affiliated Hospital of Guangxi Medical University, Nanning, Guangxi 530021, China; Department of Orthopedics, The First Affiliated Hospital of Guangxi Medical University, Nanning 530021, China

**Keywords:** electrospun nanofiber membranes, photothermal performance, anti-inflammation, antimicrobial, diabetic wound healing

## Abstract

Injury caused by excess reactive oxygen species (ROS) may lead to susceptibility to bacterial infection and sustained inflammatory response, which are the major factors impeding diabetic wound healing. By utilizing optimal anti-inflammatory, antioxidant and antibacterial biomaterials for multifunctional wound dressings is critical in clinical applications. In this study, a novel electrospun PLGA/MoS_2_@Pd nanofiber membrane was synthesized by encapsulating antioxidant and near-infrared (NIR) responsive MOS_2_@Pd nanozymes in PLGA nanofibers to form a multifunctional dressing for diabetic wound repair. With excellent biocompatibility and hemostatic ability, this novel PLGA/MoS_2_@Pd nanofiber membrane can effectively reduce oxidative stress damage and intracellular inflammatory factors expression in fibroblasts by scavenging ROS. Additionally, the PLGA/MoS_2_@Pd nanofiber membrane exhibited favorable NIR-mediated photothermal antibacterial activity *in vitro*, with inhibition rates of 97.14% and 97.07% against *Staphylococcus aureus* (*S.aureus*) and *Escherichia coli* (*E.col*i), respectively. In a diabetic rat wound infection model, NIR-assisted PLGA/MoS_2_@Pd nanofiber membrane effectively inhibited bacterial growth in the wound, reduced infection-induced inflammatory response, and promoted tissue epithelialization and collagen deposition, resulting in a wound healing rate of up to 98.5% on Day 14. This study highlighted the construction of a multifunctional nanofiber membrane platform and demonstrated its promising potential as a clinical dressing for diabetic wounds.

## Graphical abstract

**Figure:**
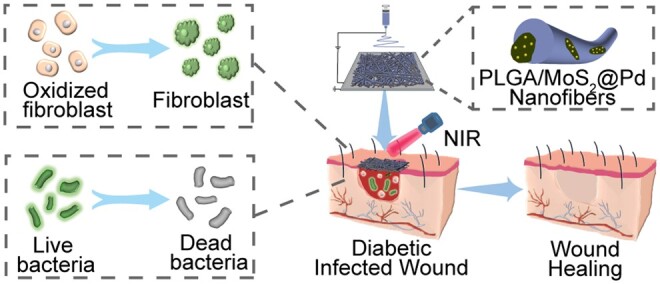

## Introduction

Diabetic wounds have become a major concern as the global diabetes prevalence increases [1, 2], which causes great physical pain and financial hardship for patients [3, 4]. Normal skin repair involves hemostasis, inflammation, proliferation and tissue remodeling [5–7]. However, accumulated reactive oxygen species (ROS) in the diabetic wound microenvironment leads to the continuous production of pro-inflammatory factors, inhibits extracellular matrix (ECM) synthesis, and delays wound healing [8–10]. Bacterial infection is another important factor contributing to impeding wound healing [11, 12]. Evidence suggests that bacterial infection and excess ROS are interrelated factors, forming a vicious cycle that continuously exacerbates wound inflammation [13]. Therefore, designing multifunctional dressings with antibacterial and ROS-scavenging properties is of great significance for wound healing [14, 15].

Electrospun nanofiber membrane have more advantages in wound repair than hydrocolloid and sponge-based conventional wound dressings due to their superior properties [16, 17]. They have an ECM-like structure that promotes cell adhesion, proliferation and migration, providing a favorable environment for soft tissue [18, 19]. Furthermore, the large surface area and high porosity of such porous electrospun nanofiber membranes can promote hemostasis for effective healing by regulating gas exchange inside and outside the dressing [20–22]. Poly(lactic-co-glycolic acid) (PLGA) is an ideal fiber matrix for electrospinning due to its high processability and controlled degradation [23–25]. More importantly, PLGA degrades into lactic and glycolic acids, which are metabolized by the body and eventually excretes, ensuring its biosafety *in vivo* [26, 27]. However, PLGA’s biological functions are insufficient to cope with the complex microenvironment in diabetic wounds. PLGA-based composite fiber membranes show great potential for photothermal therapy (PTT) and enzyme-mimicking catalysis.

In contrast to conventional antibiotic therapy, PTT employs photothermal agents that convert light energy into heat, leading to membrane rupture, protein denaturation and irreversible bacterial destruction, making it difficult to develop drug resistance [28, 29]. Molybdenum disulfide (MoS_2_) is a two-dimensional nanomaterial with excellent biocompatibility and a large specific surface area [30–32]. Its high photothermal conversion efficiency in the near infrared (NIR) range makes it an ideal candidate for antimicrobial therapy [33, 34]. On the other hand, MoS_2_ can effectively scavenge ROS through its intrinsic mimetic enzyme activity [35]. However, the limited number of active sites in MoS_2_ nanozymes limits their antioxidant performance [36]. Noble metal doping is a defective engineering strategy for improving the catalytic properties of MoS_2_ [37]. Palladium (Pd), with its distinct atomic structure, is an efficient noble metal catalyst, and the formation of S vacancies on the surface of MoS_2_ nanosheets has been reported to provide an ideal location for adsorption of Pd atoms [38, 39]. In addition, Pd nanoparticles (NPs) also exhibit photothermal, antimicrobial and ROS scavenging properties [40, 41].

**Figure 1.**
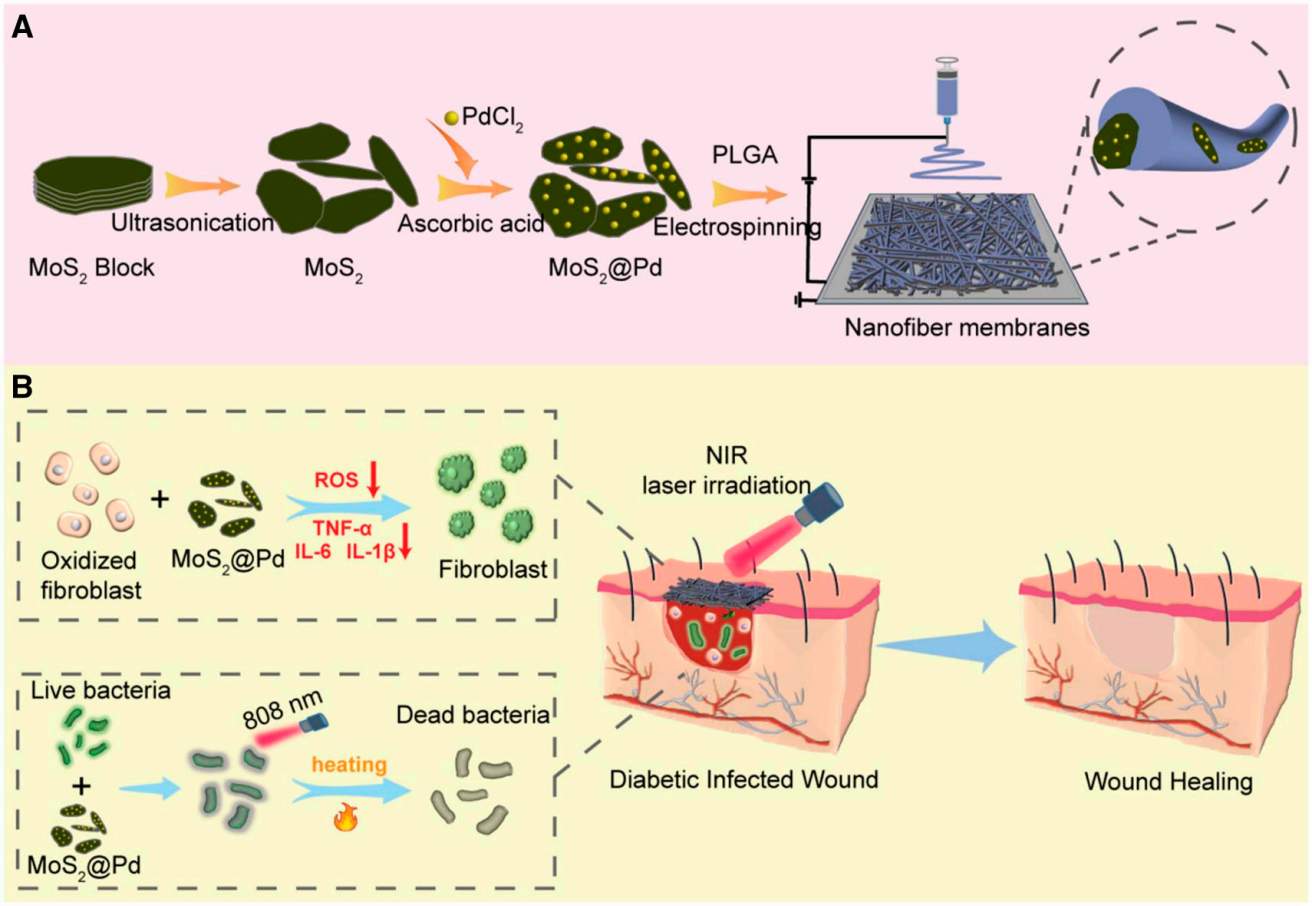
(**A**) Construction and (**B**) application of the PLGA/MoS_2_@Pd nanofiber membrane for diabetic wound healing.

Hence, we intend to prepare a novel multifunctional electrospun nanofiber membrane dressing (PLGA/MoS_2_@Pd) to promote diabetic wound healing (Figure 1), with MoS_2_@Pd as the core encapsulated in PLGA nanofibers. First, this PLGA/MoS_2_@Pd nanofiber membrane dressing has an ECM-like structure that promotes rapid hemostasis and is more conducive to cell adhesion and proliferation than conventional dressings. Second, due to the excellent photothermal properties and inherent enzyme-mimicking activity of MoS_2_ and Pd, the PLGA/MoS_2_@Pd nanofiber membrane not only has excellent antibacterial activity without drug resistance under NIR laser irradiation but also can alleviate oxidative stress by scavenging ROS, thus suppressing inflammation. Third, the evaluation of *in vivo* wound model revealed that the PLGA/MoS_2_@Pd nanofiber membrane is biocompatible and significantly promotes skin tissue regeneration and wound healing. In conclusion, the proposed multifunctional PLGA/MoS_2_@Pd nanofiber membrane may be a promising dressing for treating diabetic wounds.

## Materials and methods

### Materials

MoS_2_ (99.5% metals basis, <2 μm), carboxymethyl cellulose (DS = 0.7, 200–500 mPa.s), ascorbic acid (ACS, ≥ 99%) and palladium chloride (PdCl_2_, 99.99% metals basis) were acquired from Aladdin Chemical Reagent Co., Ltd PLGA (75:25, Mw 300 000) was purchased from Daigang Biomaterial Co., Ltd (Jinan, China). All chemical reagents were used directly with no further purification.

### Preparation of MoS_2_@Pd nanosheets

To obtain few-layer MoS_2_ nanosheets, 1 g of MoS_2_ powder was added to a mixed solution of ethanol (360 ml) and deionized water (440 ml) for 24 h of ultrasonic treatment. Then centrifuged at 5000 rpm for 20 min and freeze-dried. Next, 16 mg of MoS_2_ nanosheets were dispersed in 200 ml water, added sodium carboxymethyl cellulose (5 ml, 50 mM), which was stirred for 30 min. Ascorbic acid (1 ml, 100 mM) and PdCl_2_ (200 μl, 20 mg/ml) were added and mixing was continued for 2 h and then centrifuged at 8000 rpm for 10 min. The final product was washed and vacuum-dried to obtain MoS_2_@Pd nanosheets.

### Preparation of electrospun PLGA/MoS_2_@Pd nanofiber membranes

Briefly, 20 mg of MoS_2_@Pd nanosheets and 1 g of PLGA particles were mixed with 10 ml of hexafluoroisopropanol and agitated for 12 h. The derived mixture was then put to a 10 ml syringe for electrospinning, with the spinning head positioned 15 cm from the collector. The spinning injection speed was set to 1 mm/min, and positive pressure of 15 kV and negative pressure of 3 kV were applied. The nanofibers were collected on the collector at a speed of 100 rpm, resulting in the production of PLGA/MoS_2_@Pd nanofiber membranes. The same method was used to prepare PLGA and PLGA/MoS_2_ nanofiber membranes. Finally, nanofiber membranes were placed in a vacuum oven overnight to eliminate any remaining solvent.

### Physicochemical characterization

The morphological structure of MoS_2_@Pd and PLGA/MoS_2_@Pd were analyzed by the scanning electron microscope (SEM, VEGA3LMU, Czech Republic) and transmission electron microscope (TEM, HITACHI, H-7650). The distribution and average fiber diameter were determined using Image J software. The thickness of the nanosheets was calculated with an atomic force microscope (AFM, Bruker Dimension ICON). The samples' phase and crystallinity were evaluated using X-ray diffraction (XRD, MiniFlex600, Japan). The particle size distribution and Zeta potential of nanosheets were assessed using a particle size analyzer. The chemical composition of the nanosheets was studied using X-ray photoelectron spectroscopy (XPS, Thermo Scientific, USA). Thermal stability was evaluated using thermogravimetric analysis (TGA, NETZSCH, Germany) under N_2_ atmosphere from room temperature to 800°C. Lastly, the mechanical properties of fiber membranes were assessed through stretching by a universal material testing machine (Intalon5943, NSTRON, USA) at 10 mm/min until it broke.

### Measurement of absorption and degradation properties

A specific mass of nanofiber membrane sample was weighed and dried until a constant weight, which was recorded as the initial mass, *M*_0_. The nanofiber membrane was then immersed in deionized water and weighed at predetermined intervals, the mass was recorded as *M*_t_. The formula is: water absorption (%) = (*M*_t_ − *M*_0_)/*M*_0_ × 100%.

The initial mass of the sample in the dry state (*M*_0_) was recorded, and the sample was immersed in PBS and PBS+H_2_O_2_ solution. The samples were weighed at predetermined intervals (0, 3, 7 and 14 days) and recorded as *M*_t_. The formula is: degradation rate (%) = *M*_t_/*M*_0_ × 100%. On Day 14, the surface morphology and structural changes of the samples were observed by SEM.

### ROS scavenging ability

The ability to scavenge ROS (·OH, ·$O_{2}^{-}$ and ^1^O_2_) was detected using electron spin resonance (ESR, Bruker A300, Germany). The ESR signals were measured after treating the working solution with different fiber membranes.

### *In vitro* biocompatibility assays

#### Cell viability assay

The method used to extract and culture fibroblasts followed the guidelines established by Guangxi Medical University’s Animal Ethics Committee (approval number: 202210123). Fibroblasts were extracted from the abdominal and dorsal skin of SD rats (3–5 days). Tissues were digested with 2 mg/ml collagenase I for 2 h under 5% CO_2_ at 37°C, followed by incubation with trypsin-EDTA solution for 30 min. Fibroblasts were then cultured with DMEM medium containing 10% serum and 1% double antibody. Passage cultures were used in subsequent experiments up to the second and third generations. Cells were cultured with nanofiber membranes with a diameter of 8 mm. CCK-8 kit (CCK-8, Dojindo, Japan) was utilized to assess cytotoxicity, and the absorbance value was measured at 450 nm with a spectrophotometer (Thermo Fisher Scientific, USA).

#### Live and dead staining

Cells (2 × 10^4^ cells) were initially subjected to co-cultured with nanofiber membrane and then stained by the Calcein-AM/PI staining kit (Beyotime, China) after 1, 3 and 5 days of culture. Images were captured with a fluorescence microscope (Echo Revolve, China).

#### Cell morphology analysis

The cells cultured on the nanofiber membranes for 24 h, then fixed with a 3.7% formaldehyde solution prepared in PBS for 10–20 min and stained with a microfilament greenfluorescent probe (Actin-Tracker Red-594, Beyotime) to illustrate the cytoskeleton and DAPI (Beyotime) to stain the nuclei. The morphology of the cells growing on the nanofiber membranes was observed using a fluorescence microscope (Leica, TCS SP8).

#### Hemocompatibility evaluation

A 200 µl red blood cell suspension (5% v/v) was mixed with 800 µl of PBS and incubated with different nanofiber membranes for 4 h. A positive control of ultrapure water and a negative control of PBS were employed. The samples were observed and photographed after 4 h of incubation. The supernatant after centrifugation (2000 rpm, 5 min) was then collected and analyzed at 540 nm with a spectrophotometer (Thermo Fisher Scientific, USA).

### Hemostasis test

The hemostatic efficacy of fiber membranes was assessed by conducting a rat liver hemorrhage experiment on SD rats. The SD rats were first anesthetized before having their abdomens incised to expose their livers completely. Subsequently, to initiate bleeding, a filter paper was placed beneath the liver and punctured using a 5-ml syringe. Following this, the fiber membranes were immediately applied to the hemorrhage site of the liver. In contrast, no intervention was provided to the CON group. After 60 s, pictures were captured and recorded, and the filter paper was weighed to determine the amount of blood loss.

### Study on antioxidant and anti-inflammatory activity *in vitro*

A cellular model of oxidative stress was established using hydrogen peroxide (H_2_O_2_), with fibroblasts (2 × 10^4^) treated with H_2_O_2_ (400 μm/ml) for 24 h. Nanofiber membranes were then added, and a new medium was replaced to continue culturing for another 24 h. Intracellular ROS levels were detected using DCFH-DA (Beyotime, China) and live/dead cell staining.

The mixed cells were collected and incubated with TNF-α and IL-6 polyclonal antibodies at 4°C for 12 h, respectively. Then incubated with secondary antibody (Boster, China) (1: 100) for 1 h. Finally, the cells were stained with DAPI staining solution (Beyotime, China) for 5 min. Images were captured using a fluorescence microscope. Furthermore, inflammatory gene expression was detected by qRT-PCR. Total RNA was extracted from the treated fibroblasts using a total RNA extraction kit (Magen, China). A qPCR detection system (Roche, Germany) was used to evaluate gene expression after reverse transcription into complementary DNA (cDNA). *GAPDH* was chosen to normalize the expression levels of target genes. Table S1 provides the sequences of the primers.

### Photothermal performance

To evaluate the photothermal performance of nanofiber membranes, they were exposed to NIR laser irradiation (0.5, 1.0, 1.5 W/cm^2^) for 300 s. Photothermal images were captured, and temperature variations were recorded during the illumination process using thermal imaging camera (FOTRIC). Additionally, the photothermal stability of distinct nanofiber membranes was evaluated by exposing them to 1.5 W/cm^2^ power, which was attained by alternating 180 s of irradiation with 180 s of rest for a total of 3 cycles.

### *In vitro* antibacterial experiments

The antibacterial activity of nanofiber membranes against *Staphylococcus aureus* (*S.aureus*) and *Escherichia coli* (*E.coli*) was studied by the coating plate method. Nanofiber membranes with a diameter of 2 cm were immersed in bacterial suspension (1 ml, 1.0 × 10^7^ CFU/ml) for 4 h at 37°C. The NIR-assisted group was then exposed to 808 nm NIR laser (1.0 W/cm^2^) for 300 s. Following this, the diluted bacterial suspension (100 μl) of each group was spread on LB agar plates. The bacterial colonies were photographed and counted. In addition, the bacterial morphology was observed by SEM.

### *In vivo* wound healing assessment

Female SD rats (180–220 g) were selected for the study following the guidelines established by Guangxi Medical University’s Animal Ethics Committee (approval number: 202210123). A circular full-thickness skin wound with a diameter of 20 mm was created on the rat's back. Various nanofiber membranes were applied to the wound. The wound was exposed to 808 nm NIR laser at a power density of 1 W/cm^2^ for 300 s, and temperature changes were recorded with a thermal image.

Streptozotocin (STZ, 1%) was used to establish a diabetic rat model. Subsequently, 20 μl of *S.aureus* (1.0 × 10^7^ CFU/ml) was evenly distributed over the wound surface for 1 day. Infected diabetic rats were randomly divided into six groups for different treatments: CON, PLGA, PLGA/MoS_2_, PLGA/MoS_2_@Pd, PLGA/MoS_2_+NIR and PLGA/MoS_2_@Pd+NIR. Wounds were measured and photographed on Days 0, 3, 7 and 14 to assess wound repair. Additionally, wound exudate from Day 1 was collected and serially diluted with PBS before being spread on agar plates. H&E staining and Masson staining were used to assess the extent of wound regeneration and collagen deposition, respectively. Additionally, immunohistochemical (TNF-α and IL-6) staining was used to assess the inflammatory response.

### Statistical analysis

All means were statistically compared using GraphPad Prism 8 (GraphPad, USA). One-way ANOVA was used to perform multiple comparisons. *P *≤* *0.05 (* or #), *P *≤* *0.01 (** or ##) and *P *≤* *0.001 (*** or ###) means statistically significant, while “ns” denotes not significant.

## Results and discussion

### Fabrication and characterization of nanosheets

**Figure 2.**
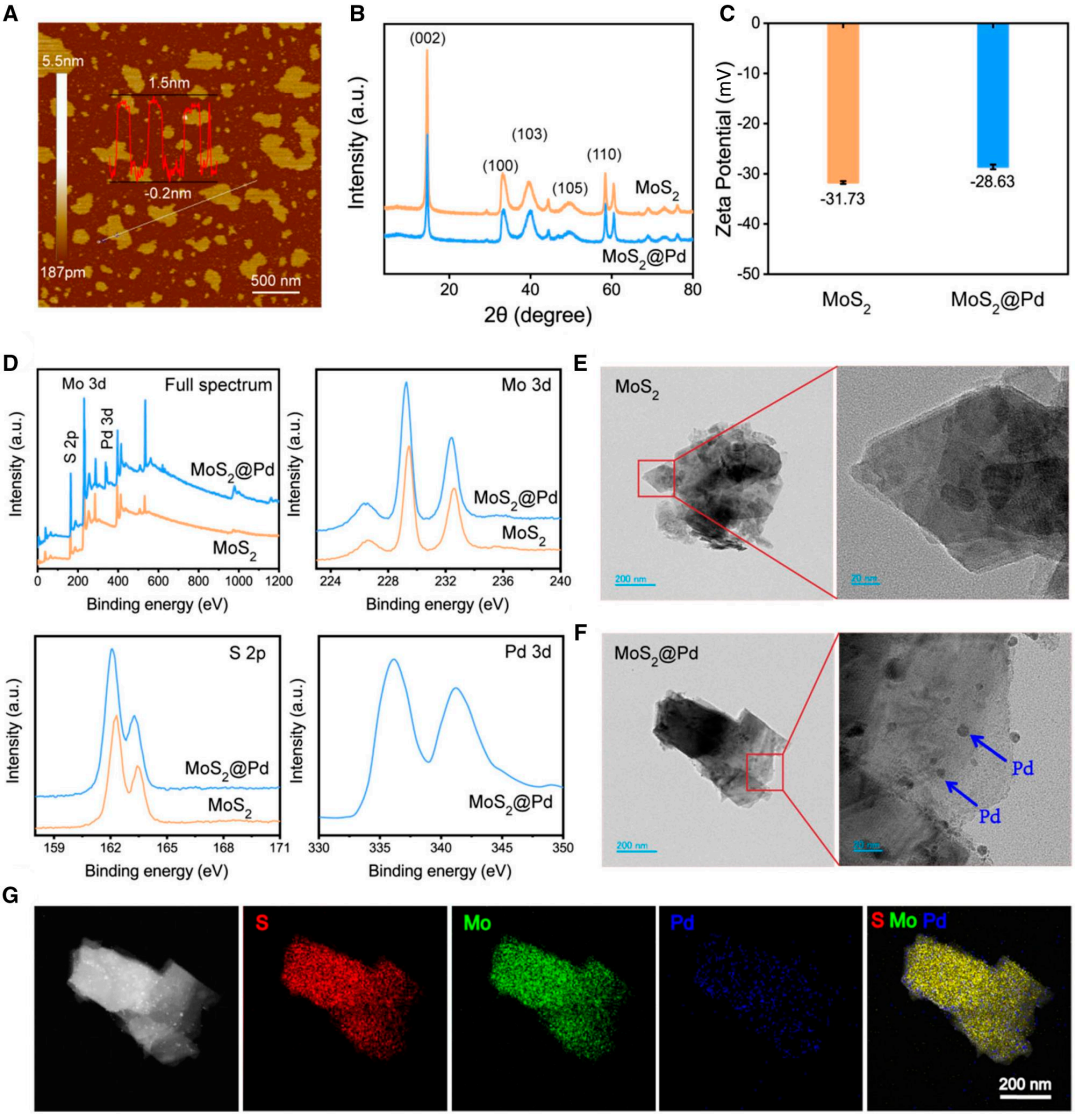
Characterization of MoS_2_@Pd. (**A**) AFM images of MoS_2_ nanosheets; (**B**) XRD patterns of MoS_2_ and MoS_2_@Pd; (**C**) zeta potential of MoS_2_ and MoS_2_@Pd; (**D**) XPS results of MoS_2_ and MoS_2_@Pd; TEM images of (**E**) MoS_2_ and (**F**) MoS_2_@Pd; (**G**) corresponding elemental mappings of S, Mo and Pd in MoS_2_@Pd.

MoS_2_ nanosheets were produced by mechanically exfoliating bulk MoS_2_ and loaded with Pd NPs via ascorbic acid reduction, obtaining MoS_2_@Pd. The AFM measurements indicated that the thickness of MoS_2_ nanosheets was predominantly distributed at approximately 1.5 nm, corresponding to a few layers of MoS_2_ (Figure 2A). The crystallization and phase composition of MoS_2_ and MoS_2_@Pd were analyzed using XRD spectroscopy (Figure 2B). Following exfoliation, MoS_2_ exhibited characteristic peaks at (002), (100), (103), (105) and (110) crystal planes, indicating its layered 2H crystal phase [42]. The XRD spectrum of MoS_2_@Pd showed no significant differences from that of MoS_2_, probably due to the ultra-small size of Pd loaded onto MoS_2_. Zeta potential (Figure 2C) of MoS_2_ and MoS_2_@Pd were determined to be −31.73 mV and −28.63 mV, respectively. The slightly altered potential may be attributed to the positively charged Pd modification [43]. The particle size distribution of MoS_2_@Pd was slightly larger than that of MoS_2_, as observed in Figure S1. XPS was employed to detect their elemental compositions (Figure 2D), revealing molybdenum (Mo) and sulfur (S) spectra in both MoS_2_ and MoS_2_@Pd, but only MoS_2_@Pd exhibited Pd spectra, indicating successful loading of Pd onto MoS_2_. The morphological structures were analyzed via TEM (Figure 2E and F), which revealed an irregular layered structure for MoS_2_ and ultra-small Pd NPs uniformly dispersed on the surface of MoS_2_@Pd. The elemental image of MoS_2_@Pd exhibited obvious Pd elements compared to MoS_2_ (Figure 2G and Figure S2), further confirming that Pd was successfully loaded onto MoS_2_. The provided data (Table S2) presented the elemental composition of MoS_2_ and MoS_2_@Pd NPs, focusing on the percentage of S, Mo, and Pd determined by Energy Dispersive X-ray (EDX) analysis. The ratio of Pd in MoS_2_@Pd was 5.34% (±1.71), indicating successful deposition of Pd on the MoS_2_ surface.

### Fabrication and characterization of nanofiber membranes

**Figure 3.**
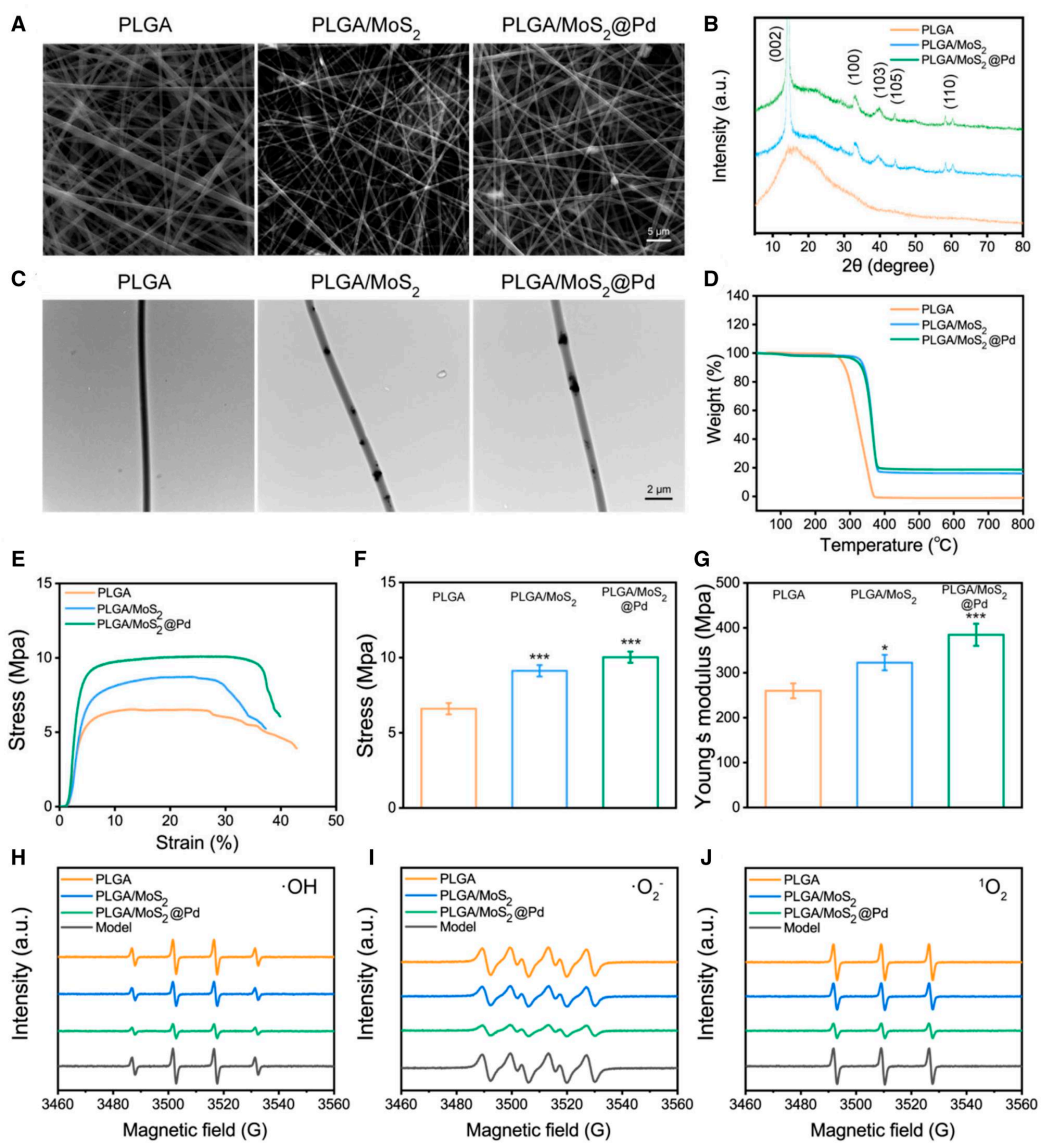
Fabrication and characterization of nanofiber membranes. (**A**) SEM and (**B**) TEM images of PLGA, PLGA/MoS_2_, and PLGA/MoS_2_@Pd; (**C**) XRD patterns and (**D**) TGA curves of PLGA, PLGA/MoS_2_ and PLGA/MoS_2_@Pd; (**E**–**G**) mechanical properties of PLGA, PLGA/MoS_2_, and PLGA/MoS_2_@Pd; (**H**) ·OH, (**I**) ·$O_{2}^{-}$ and (**J**) ^1^O_2_ scavenging ability measured by ESR.

We utilized the electrospinning technique to fabricate nanofiber membranes. The morphology and structure of PLGA, PLGA/MoS_2_ and PLGA/MoS_2_@Pd were then examined using SEM and TEM. Figure S3 and Figure 3A showed SEM images of PLGA/MoS_2_@Pd nanofiber membranes containing 1%, 2% and 4% MoS_2_@Pd. The 1% and 2% MoS_2_@Pd nanofiber membranes were composed of continuous smooth fibers that were interwoven to form an ECM-like porous network structure. However, a higher concentration of MoS_2_@Pd (4%) caused noticeable aggregation and incomplete encapsulation by PLGA. To ensure structural stability, PLGA/MoS_2_@Pd nanofiber membranes containing 2% MoS_2_@Pd were chosen for the subsequent experiments. The TEM results revealed that MoS_2_ and MoS_2_@Pd nanosheets were embedded uniformly in the fibers (Figure 3B). Figure S4 showed that the average fiber diameters for PLGA, PLGA/MoS_2_ and PLGA/MoS_2_@Pd were 661 ± 233, 491 ± 137 and 564 ± 164 nm, respectively. Compared to PLGA, the fiber diameters of PLGA/MoS_2_ and PLGA/MoS_2_@Pd were smaller and exhibited a narrower distribution. This is probably due to the addition of MoS_2_, which improved the conductivity of the electrospinning solution, resulting in more uniform fibers. Figure 3C showed the XRD patterns of PLGA, PLGA/MoS_2_, and PLGA/MoS_2_@Pd. Both PLGA/MoS_2_ and PLGA/MoS_2_@Pd nanofiber membranes retained the fine crystal structure of the MoS_2_ NPs. The peaks correspond to the (002), (100) (103), (105) and (110) reflections of MoS_2_, respectively. The XRD spectra of PLGA/MoS_2_@Pd are not significantly different from those of PLGA/MoS_2_, which may be attributed to the formation of ultra-small Pd NPs and the low crystallinity of Pd embedded in the nanofiber membranes.

The thermal behavior of nanofiber membranes was investigated using TGA [44]. As demonstrated in Figure 3D, weight loss occurred around 250°C due to the thermal decomposition of PLGA. However, compared to PLGA, PLGA/MoS_2_ and PLGA/MoS_2_@Pd lost weight at slightly higher temperatures and produced remnants of nanomaterials. These results indicated that the presence of MoS_2_ improved the thermal stability of PLGA/MoS_2_ and PLGA/MoS_2_@Pd [45]. The tensile stress-strain curves of different nanofiber membranes were presented in Figure 3E. Tensile stresses for PLGA, PLGA/MoS_2_ and PLGA/MoS_2_@Pd were approximately 6.60 ± 0.38, 9.12 ± 0.39 and 10.03 ± 0.37 MPa (Figure 3F), respectively. Additionally, as shown in Figure 3G, Young’s modulus values were approximately 259.77 ± 16.66, 322.64 ± 17.28 and 384.50 ± 24.61 MPa for PLGA, PLGA/MoS_2_, and PLGA/MoS_2_@Pd, respectively. Mechanical tests revealed that PLGA/MoS_2_ and PLGA/MoS_2_@Pd had higher mechanical properties than PLGA, indicating that adding MoS_2_ and MoS_2_@Pd improved their ability to withstand external stresses, such as friction, stretching, and compression [46, 47]. This enhanced durability helps prevent displacement and maintain the structural integrity of the dressing, which is crucial for providing continuous protection and reducing the risk of infection during the healing process. The ability to scavenge ROS was evaluated using ESR spectroscopy [48]. Figure 3H–J demonstrated that the scavenging effect of PLGA nanofiber membrane was not significant for ·OH, ·$O_{2}^{-}$ and ^1^O_2_ radicals, while PLGA/MoS_2_ nanofiber membrane had some scavenging ability. The scavenging effect of PLGA/MoS_2_@Pd nanofiber membranes on ·OH, ·$O_{2}^{-}$ and ^1^O_2_ radicals with the strongest scavenging ability. These results indicated that PLGA/MoS_2_@Pd nanofiber membrane had a significantly higher ROS scavenging ability.

In addition, Figure S5 showed that PLGA/MoS_2_ and PLGA/MoS_2_@Pd membranes had significantly higher porosity than pure PLGA membrane. This enhancement may be attributed to the introduction of MoS_2_ and MoS_2_@Pd, which affects the fiber structure and reduces the fiber diameters of PLGA/MoS_2_ and PLGA/MoS_2_@Pd membranes, resulting in increased porosity. Higher porosity promotes better gas exchange and nutrient penetration, providing a more favorable environment for cell growth, particularly in supporting cell proliferation and tissue repair during wound healing [49, 50]. Figure S6 demonstrated that PLGA/MoS_2_ and PLGA/MoS_2_@Pd nanofiber membranes had significantly higher water absorption capacity than pure PLGA nanofiber membrane. Figure S7 demonstrated the degradation rates of PLGA and composites were relatively slow in PBS, with the residual mass remaining above 90% after 14 days, whereas in H_2_O_2,_ the residual mass decreased to around 80%. Figure S8 showed that three membranes treated with PBS still had a relatively intact nanofiber structure. Despite some slight fiber thinning, the overall network structure remained intact. After H_2_O_2_ treatment, PLGA, PLGA/MoS_2_ and PLGA/MoS_2_@Pd nanofiber membranes showed different degrees of fracture and shrinkage, suggesting that the degradation of the PLGA-based materials in the H_2_O_2_ environment is faster than that in the PBS environment. The faster degradation of PLGA-based composites in oxidative environments is beneficial for releasing active components in these specific environments, thereby enhancing the therapeutic effects of the materials.

### *In vitro* biocompatibility and hemostatic ability

**Figure 4.**
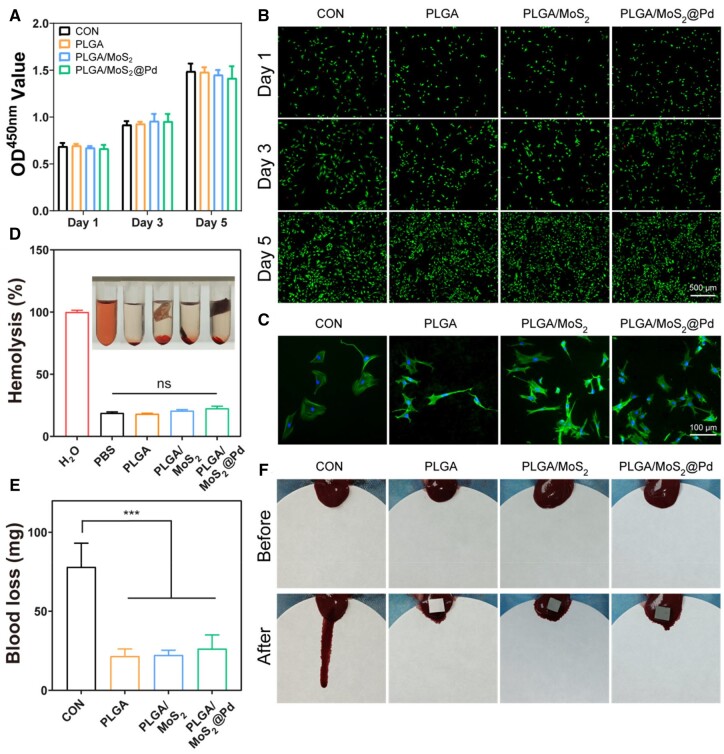
*In vitro* biocompatibility and hemostatic ability of nanofiber membranes. (**A**) CCK-8 results after exposure to different nanofiber membranes (*n *=* *3); (**B**) cell staining results of fibroblasts after different treatments; (**C**) cytoskeleton staining of fibroblasts cultured on different specimens on Day 1; (**D**) hemocompatibility of PLGA, PLGA/MoS_2_ and PLGA/MoS_2_@Pd nanofiber membranes. (*n *=* *3); (**E**) blood loss of nanofiber membrane in the rat hemorrhaging liver model (*n *=* *3, and *** means *P *<* *0.001); (**F**) photographs displaying nanofiber membrane after application to the rat hemorrhaging liver model.

Biocompatibility is a critical factor for the successful application of biomaterials [51]. Figure S9 illustrated the effect of PLGA/MoS_2_@Pd nanofiber membrane with varying MoS_2_@Pd concentrations on cell viability. The results indicated that at lower MoS_2_@Pd concentrations (1% and 2%), the relative cell viability of PLGA/MoS_2_@Pd group was close to that of the CON group, suggesting excellent biocompatibility. However, at 4% MoS_2_@Pd content, the cell viability decreased significantly. Therefore, PLGA/MoS_2_@Pd nanofiber membrane with 2% MoS_2_@Pd were chosen for the following experiments. The CCK-8 assay was employed to assess the effect of PLGA/MoS_2_@Pd on cell proliferation. The results (Figure 4A) revealed that PLGA/MoS_2_@Pd nanofiber membrane had no significant cytotoxicity. Subsequently, the live/dead cell staining (Figure 4B) showed that there were only a few dead cells in all groups and no difference in cell viability. Cytoskeleton staining was used to examine cell morphology after 24 h of co-culture with nanofiber membranes (Figure 4C). Cells cultured on nanofiber membranes spread rapidly, and most of the cells had an ideal spindle shape, with more pseudopodia than the CON group. This is because nanofiber membranes mimicked the structure of ECM, resulting in improved cell adhesion and confirming nanofiber membranes' excellent cytocompatibility [52].

Next, we evaluated the hemocompatibility of nanofiber membranes. As shown in Figure 4D, red blood cells treated with deionized water appeared red due to rupture, whereas cells treated with PBS and nanofiber membranes were mostly clear. There was no significant difference in optical density (OD) values between nanofiber membranes and PBS, indicating that these nanofiber membranes had good blood compatibility. Furthermore, the prepared nanofiber membranes demonstrated excellent hemostatic properties (Figure 4E and F). The CON group lost significantly more blood (78 mg) than the PLGA, PLGA/MoS_2_ and PLGA/MoS_2_@Pd groups (21.67, 22.33 and 26.33 mg, respectively). In conclusion, the designed PLGA/MoS_2_@Pd nanofiber membrane exhibited excellent biocompatibility and hemostatic ability, making it safe for using in skin wounds.

### *In vitro* antioxidant and anti-inflammatory effect

**Figure 5.**
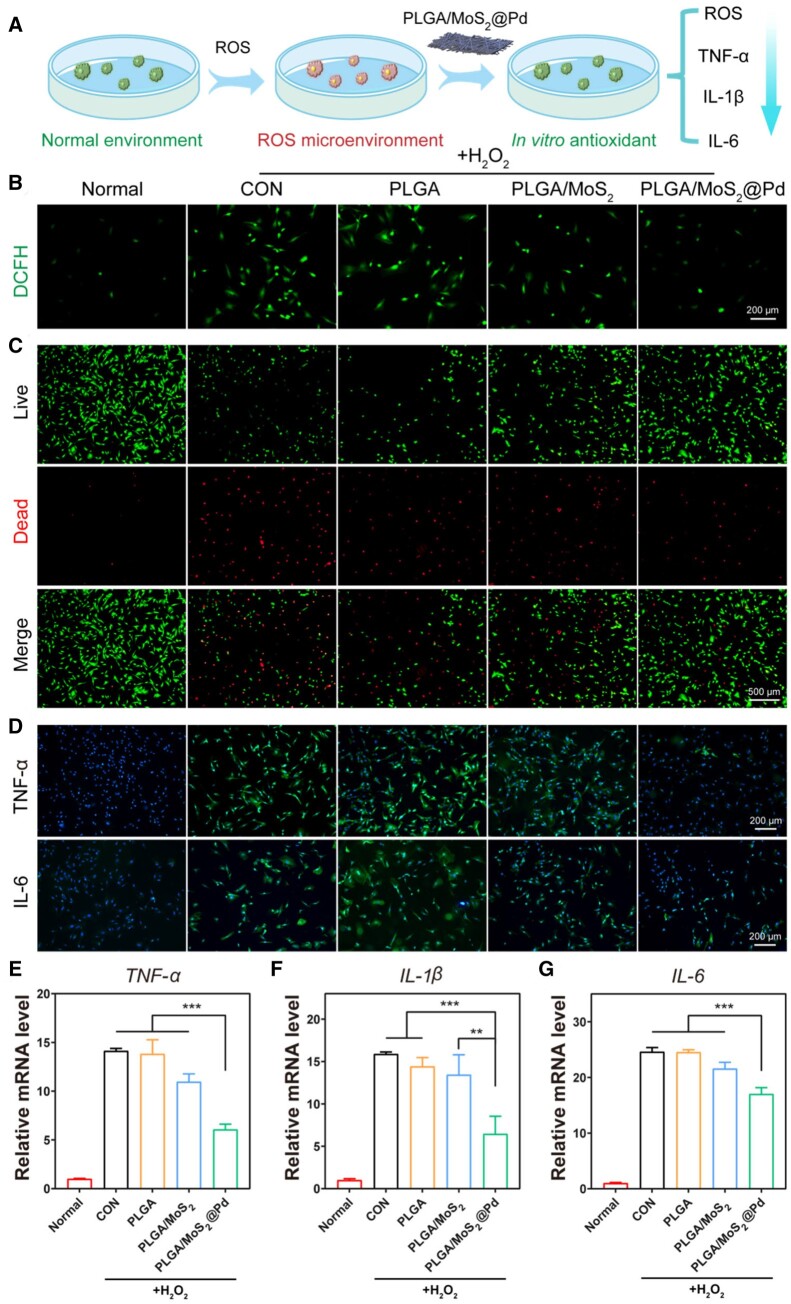
*In vitro* antioxidant and anti-inflammatory effect of PLGA/MoS_2_@Pd nanofiber membrane. (**A**) A schematic diagram of the action of the PLGA/MoS_2_@Pd nanofiber membrane; (**B**) fluorescence results describing the ability of nanofiber membranes to scavenge ROS; (**C**) representative fluorescence images of H_2_O_2_-induced fibroblasts death after different treatments; (**D**) detection of inflammatory factors (IL-6 and TNF-α) in fibroblasts by immunofluorescence staining; (**E**) *TNF-α*, (**F**) *IL-1β* and (**G**) *IL-6* by qRT-PCR.

Given their strong ROS scavenging ability, we evaluated the protective effect of PLGA/MoS_2_@Pd on ROS-induced oxidative damage in a cell model (Figure 5A). DCFH was used to assess the capacity of PLGA/MoS_2_@Pd to remove ROS at the cellular level [53]. Figure 5B and Figure S10 showed that H_2_O_2_ stimulation (CON group) significantly increased fluorescence intensity in cells, indicating that ROS levels were higher in H_2_O_2_-induced cells than in normal fibroblast (Normal group). However, the PLGA/MoS_2_@Pd group showed significantly lower intracellular ROS levels than the CON group, indicating the significant antioxidant effect of PLGA/MoS_2_@Pd nanofiber membrane. Live/dead cell staining was used to measure cell viability following exposure to an oxidative microenvironment. Figure 5C and Figure S11 showed that only half of the cells in the CON group survived at a concentration of 400 μM H_2_O_2_. However, in the PLGA/MoS_2_ group with the same amount of H_2_O_2_, cell viability increased slightly to 66%, whereas in the PLGA/MoS_2_@Pd group, dead cells were significantly reduced and relative cell viability significantly increased to 84%. These results indicated that the PLGA/MoS_2_@Pd nanofiber membrane could scavenge ROS in cells and protect them from oxidative damage.

The anti-inflammatory effect of PLGA/MoS_2_@Pd nanofiber membrane is attributed to its antioxidant properties, which effectively regulate the inflammatory environment during wound healing. Immunofluorescence staining was performed to observe the expression of inflammatory factors in H_2_O_2_-induced fibroblasts [54]. As shown in Figure 5D and Figure S12, fibroblasts stimulated with H_2_O_2_ (CON group) exhibited significantly increased fluorescence intensity, indicating high positive expression of TNF-α and IL-6 compared to normal fibroblasts (Normal group). In contrast, the PLGA/MoS_2_ and PLGA/MoS_2_@Pd groups showed a decrease in TNF-α expression to 79.45% and 43.34%, respectively. Similarly, the expression of IL-6 was significantly reduced to 55.24% in the PLGA/MoS_2_@Pd group, indicating the most effective inhibition of inflammatory factor expression.

qRT-PCR was used to further investigate the anti-inflammatory activity of PLGA/MoS_2_@Pd nanofiber membrane. As depicted in Figure 5E–G, inflammatory markers were low in the Normal group, However, after H_2_O_2_ stimulation, inflammatory factors (*TNF-α*, *IL-1β*, *IL-6*) were significantly upregulated to 14.12, 15.89 and 24.60, respectively. The PLGA/MoS_2_@Pd group significantly reduced expression levels (6.078, 6.460 and 17.02, respectively). These findings demonstrated that PLGA/MoS_2_@Pd exhibited substantial antioxidant and anti-inflammatory effects on H_2_O_2_-treated fibroblasts. Moreover, compared to MoS_2_ nanosheets alone, MoS_2_@Pd enhanced the antioxidant and anti-inflammatory abilities of cells.

### *In vitro* photothermal and antibacterial performance

**Figure 6.**
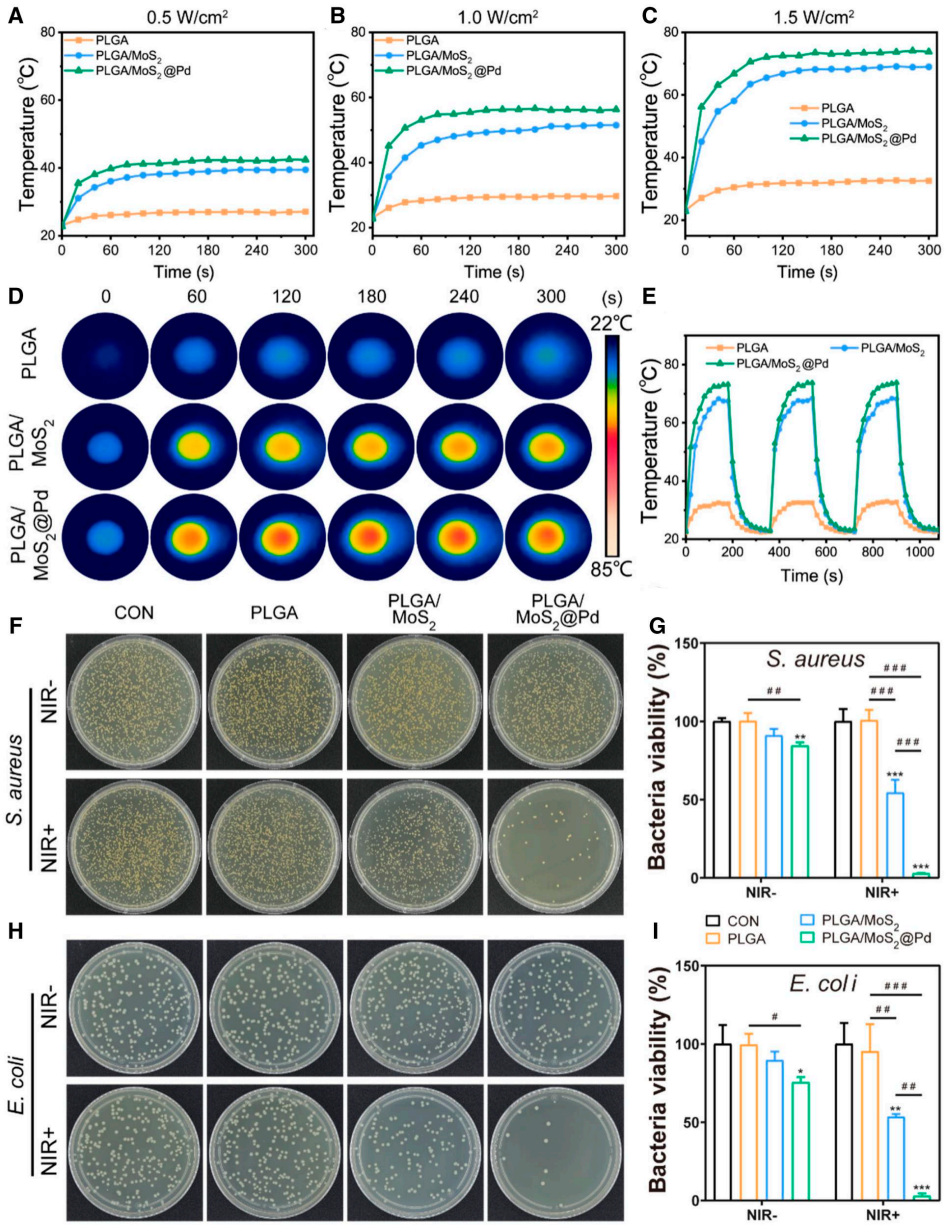
*In vitro* photothermal and antibacterial performance. Photothermal curves for nanofiber membranes under 808 nm NIR laser irradiation at power densities of (**A**) 0.5 W/cm^2^, (**B**) 1.0 W/cm^2^ and (**C**) 1.5 W/cm^2^; (**D**) infrared thermal images of nanofiber membrane under 808 nm NIR irradiation (1.0 W/cm^2^); (**E**) photothermal stability under repeated irradiation (1.0 W/cm^2^); (**F**) photographs of *S. aureus* bacterial colonies and (**G**) quantitative analysis of relative bacterial survival rate; (**H**) photographs of *E.coli* bacterial colonies and (**I**) quantitative analysis of relative bacterial survival rate after different treatments.

The photothermal properties of various nanofiber membranes were assessed by recording temperature changes with NIR laser irradiation (0.5, 1.0 and 1.5 W/cm^2^) [55, 56]. As depicted in Figure 6A, at NIR irradiation of 0.5 W/cm^2^, PLGA/MoS_2_ and PLGA/MoS_2_@Pd nanofiber membranes reached 39.5°C and 42.4°C from their initial temperatures of 23.3°C and 22.7°C, respectively. However, the temperature of PLGA remained consistent. Figure 6B and C revealed that as the power increased, the temperature of PLGA/MoS_2_ rose to 51.5°C (1.0 W/cm^2^) and 69°C (1.5 W/cm^2^), while that of PLGA/MoS_2_@Pd rose to 56.3°C (1.0 W/cm^2^) and 73.8°C (1.5 W/cm^2^). In contrast, PLGA had only a slight temperature change. The thermal images (Figure 6D and Figures S13 and S14) also demonstrated that PLGA/MoS_2_@Pd had better photothermal performance than PLGA/MoS_2_. Additionally, Figure 6E illustrated that the temperature of the PLGA/MoS_2_@Pd nanofiber membrane remained almost constant after three cycles of irradiation. These results indicated that the designed PLGA/MoS_2_@Pd could rapidly convert 808 nm NIR light into thermal energy with excellent photothermal performance and photothermal stability, making it a promising photothermal platform for antibacterial therapy.

The PLGA/MoS_2_@Pd nanofiber membrane prepared in this study demonstrated outstanding photothermal conversion performance, prompting us to investigate its antibacterial properties. Figure 6F and H showed that a large number of colonies appeared on LB plates in both the CON group with and without the NIR laser irradiation, indicating that NIR irradiation alone did not affect the growth of *E.coli* and *S.aureus*. However, the colony counts of *S.aureus* and *E.coli* in the PLGA/MoS_2_ +NIR group decreased significantly to 54.46% and 53.45%, respectively, when compared to PLGA/MoS_2_ without NIR laser irradiation (Figure 6G and I). In addition, the loading of Pd NPs endowed the PLGA/MoS_2_ nanofiber membrane with higher antimicrobial capacity, with inhibition rates of 97.14% and 97.07% against *S.aureus* and *E.coli* in the PLGA/MoS_2_@Pd+NIR group, respectively. We further evaluated the effect of different MoS_2_@Pd contents on the antimicrobial activities of PLGA/MoS_2_@Pd nanofiber membrane. The antibacterial performance of PLGA/MoS_2_@Pd improved as the MoS_2_@Pd concentration increased (Figure S15). Particularly, PLGA/MoS_2_@Pd substantially inhibited the growth of bacteria under NIR laser irradiation, approaching the commercially available 3M antimicrobial dressing. This suggests that PLGA/MoS_2_@Pd nanofiber membrane, through a combination of photothermal effects and the inherent antimicrobial properties of MoS_2_@Pd, has significant potential for antimicrobial applications, particularly in treating wound infections.

Subsequently, the bacterial morphology was examined using SEM after various treatments (Figures S16 and S17). The results showed that *E.coli* and *S.aureus* in PLGA, PLGA/MoS_2_, and PLGA/MoS_2_@Pd remained spherical and rod-shaped with smooth surfaces without NIR irradiation. In contrast, after 300 s of NIR laser irradiation, the bacterial morphology of the PLGA+NIR group was close to that of the CON group. The PLGA/MoS_2_+NIR group exhibited some shrinkage and stomata, while the PLGA/MoS_2_@Pd+NIR group showed more severe damage, including deformation and even rupture of bacterial membranes. These findings suggests that the PLGA/MoS_2_@Pd nanofiber membrane had the strongest bactericidal effect under NIR laser irradiation, effectively destroying the bacterial membrane.

### *In vivo* assessment of wound healing performance

**Figure 7.**
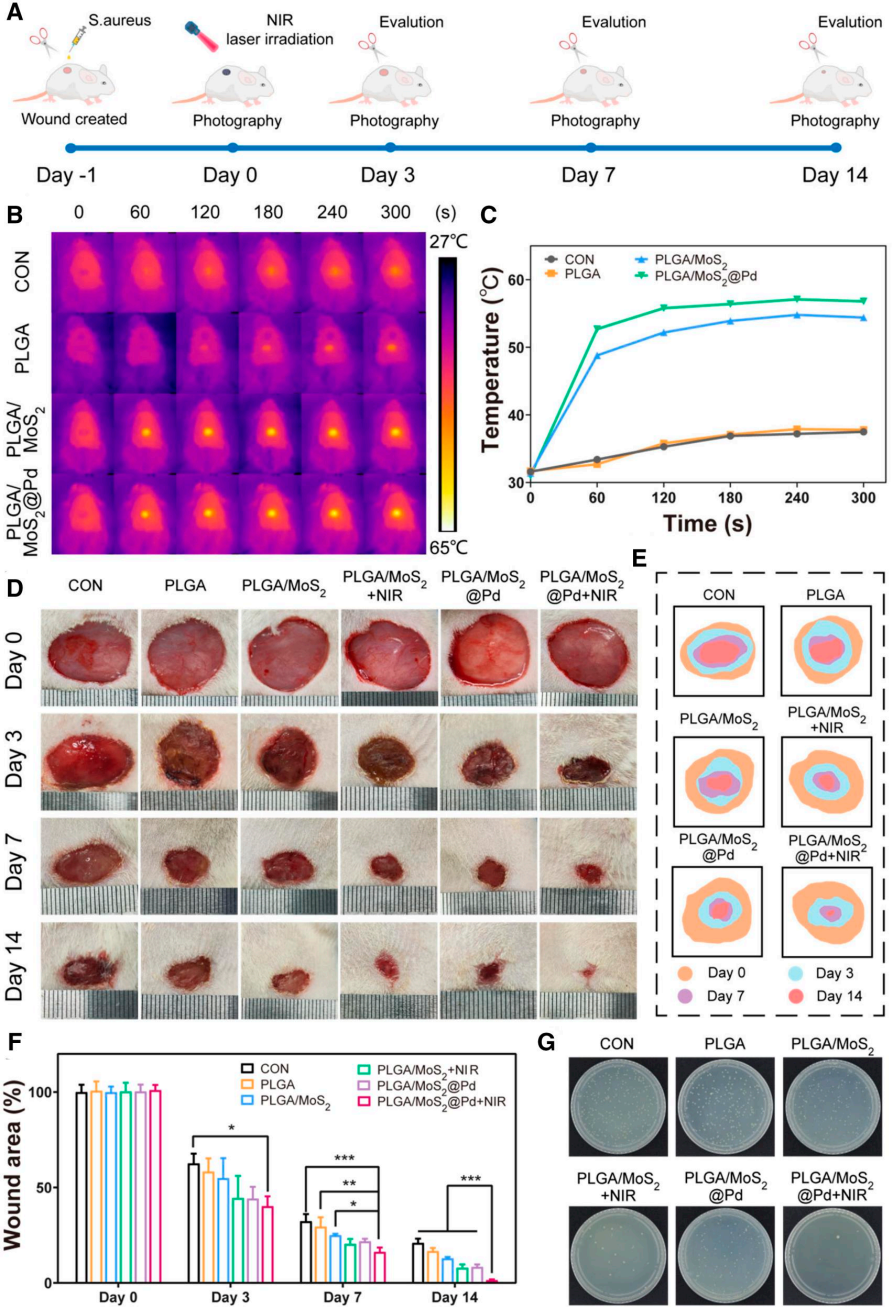
*In vivo* assessment of wound healing performance. (**A**) Demonstration of constructing infected wounds and healing process; (**B**) infrared thermal images and (**C**) thermal curves of infected wounds treated with different treatments after NIR irradiation (1.0 W/cm^2^); (**D**) representative images, (**E**) schematic images and (**F**) the wound closure rates under different treatments on Days 0, 3, 7 and 14; (**G**) photographs of bacterial colonies at wounds on Day 1 after different treatments.

Encouraged by the remarkable bacterial inhibitory, antioxidant, and anti-inflammatory abilities of the PLGA/MoS_2_@Pd nanofiber membrane, we proceeded to evaluate its *in vivo* wound-healing potential in a bacterial-infected diabetic rat skin wound model (Figure 7A). Firstly, we assessed the photothermal effect of the membranes *in vivo* using thermal imaging (Figure 7B and C). After 300 s of irradiation with an 808 nm NIR laser (1.0 W/cm^2^), the temperature of rats in the CON and PLGA groups changed only slightly, whereas the PLGA/MoS_2_@Pd+NIR group experienced a rapid increase in temperature from 31°C to 57°C. This observation demonstrated that the PLGA/MoS_2_@Pd nanofiber membrane maintained its excellent photothermal properties *in vivo*. We further evaluated the safety of the NIR laser irradiation (1.0 W/cm^2^). Figure S18A showed no significant differences in rat skin before and after irradiation for 0 h and 24 h (black circles indicate the irradiated areas), confirming that the temperature increase induced by the NIR laser irradiation did not cause visible skin damage. Additionally, Figure S18B and C demonstrated that the skin structure remained normal in all groups. This further suggests that the temperature reached during NIR treatment was maintained within a safe range, without causing harm to the skin.

Since wounds treated with PBS and PLGA nanofiber membranes exhibited no significant temperature change following NIR irradiation, the subsequent animal experiments were divided into six groups: CON, PLGA, PLGA/MoS_2_, PLGA/MoS_2_+NIR, PLGA/MoS_2_@Pd and PLGA/MoS_2_@Pd+NIR. Figure S19 presented the macroscopic images of the wound models. The wound healing process images (Figure 7D), schematic diagrams (Figure 7E) and quantitative analyses of the wound area (Figure 7F) showed that on Day 3, the wounds in the CON group exhibited exudate, whereas the wounds in the other nanofiber membrane groups displayed varying degrees of scab formation. This is due to the absorbent nature of the nanofiber membrane, which made the wounds relatively dry. On Day 3, wounds treated with PLGA/MoS_2_@Pd+NIR were significantly different from the CON group, and this advantage became increasingly apparent over time. On Day 14, the wounds treated with PLGA/MoS_2_@Pd+NIR showed the smallest area (1.437%) which was mostly covered by newly formed skin. In contrast, the CON group and the groups treated with PLGA, PLGA/MoS_2_, PLGA/MoS_2_+NIR and PLGA/MoS_2_@Pd showed wound areas of 20.98%, 16.67%, 12.88%, 7.926% and 8.400%, respectively. These findings indicated that PLGA/MoS_2_@Pd nanofiber membrane could significantly accelerate wound healing, and the combination of PLGA/MoS_2_@Pd and NIR had the best healing effect. Additionally, bacterial plate count [57] results (Figure 7G) showed PLGA/MoS_2_@Pd+NIR group had the least amount of bacteria, consistent with the *in vitro* antibacterial results.

### Histological changes in regenerated skin

**Figure 8.**
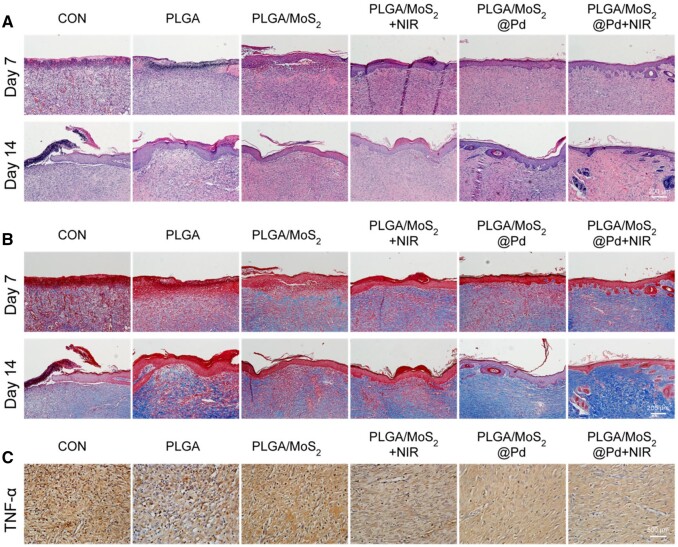
Histological staining of wounds. (**A**) H&E staining and (**B**) Masson staining of wounds on Days 7 and 14 post-operation; (**C**) immunohistochemistry images for TNF-α after different treatments on Day 14 post-operation.

Finally, H&E staining was used to assess the wound healing process. Figure 8A revealed that both PLGA/MoS_2_@Pd and PLGA/MoS_2_@Pd+NIR groups exhibited epithelialization after 7 days of treatment. Notably, the PLGA/MoS_2_@Pd+NIR group showed the presence of newly regenerated hair follicles, highlighting the beneficial effects of the combined treatment. On Day 14, epithelial remodeling remained incomplete in the CON group, whereas the PLGA/MoS_2_@Pd+NIR group displayed an increase in epithelial hair follicles, which had normalized, indicating more effective tissue regeneration. Furthermore, Masson staining was used to assess collagen deposition. Figure 8B demonstrated that at Day 7 and 14, the PLGA/MoS_2_@Pd+NIR group had significantly more collagen deposition than the other groups, which is more favorable for ECM synthesis and skin regeneration. The above results confirmed the advantage of NIR-assisted PLGA/MoS_2_@Pd fiber membrane in wound healing. Additionally, immunohistochemical analysis revealed the expression levels of inflammatory markers TNF-α and IL-6 (Figure 8C and Figure S20). The expression levels of TNF-α and IL-6 were significantly lower in the PLGA/MoS_2_@Pd+NIR group, followed by the PLGA/MoS_2_@Pd group. All of these results suggest that PLGA/MoS_2_@Pd nanofiber membranes can reduce inflammation, promote epithelialization and collagen deposition. More importantly, these effects were further enhanced under NIR irradiation, resulting in improved wound repair. Moreover, H&E staining of key organs in all groups showed no signs of damage (Figure S21), confirming that the PLGA/MoS_2_@Pd nanofiber membrane is biologically safe for *in vivo* use. These findings are consistent with *in vitro* studies, further supporting the therapeutic potential of PLGA/MoS_2_@Pd nanofiber membrane in clinical applications.

## Conclusions

In this study, we successfully developed a biocompatible nanofiber membrane dressing made of PLGA/MoS_2_@Pd for diabetic wounds. This dressing combined antioxidant, anti-inflammatory and antibacterial properties to optimize therapeutic efficacy. The PLGA/MoS_2_@Pd nanofiber membrane could eliminate excess ROS, relieve oxidative stress damage, and reduce inflammatory factors, thus promoting wound healing. Additionally, the nanofiber membrane had a high photothermal conversion rate, providing efficient bactericidal action through the combination of intrinsic antibacterial activity and photothermal bacterial ablation. In the infected wound model, the NIR-assisted PLGA/MoS_2_@Pd nanofiber membrane effectively eliminated bacterial infection, decreased infection-induced inflammation, promoted epithelialization and collagen deposition and accelerated diabetic wound healing. In summary, the multi-active PLGA/MoS_2_@Pd nanofiber membrane dressing proposed in this study has significant clinical potential for treating diabetic wounds. However, the antibacterial effect and mechanism of PLGA/MoS_2_@Pd nanofiber membrane combined with PTT on methicillin-resistant Staphylococcus aureus (MRSA) infections is worth further exploration.

## Supplementary Material

rbae143_Supplementary_Data

## Data Availability

The raw data and processed data required to reproduce these findings are available from the corresponding author upon request.

## References

[1] Li G , Wang Q, Feng J, Wang J, Wang Y, Huang X, Shao T, Deng X, Cao Y, Zhou M, Zhao C. Recent insights into the role of defensins in diabetic wound healing. Biomed Pharmacother 2022;155:113694. 36099789 10.1016/j.biopha.2022.113694

[2] Li P , Hong G, Zhan W, Deng M, Tu C, Wei J, Lin H. Endothelial progenitor cell derived exosomes mediated miR-182-5p delivery accelerate diabetic wound healing via down-regulating PPARG. Int J Med Sci 2023;20:468–81. 37057206 10.7150/ijms.78790 PMC10087624

[3] Chao D , Dong Q, Yu Z, Qi D, Li M, Xu L, Liu L, Fang Y, Dong S. Specific nanodrug for diabetic chronic wounds based on antioxidase-mimicking MOF-818 nanozymes. J Am Chem Soc 2022;144:23438–47. 36512736 10.1021/jacs.2c09663

[4] Song J , Zhang YN, Chan SY, Du ZY, Yan YJ, Wang TJ, Li P, Huang W. Hydrogel-based flexible materials for diabetes diagnosis, treatment, and management. NPJ Flex Electron 2021;5:26.

[5] Huang Y , Ding Y, Wang B, Ji Q, Peng C, Tan Q. Neutrophils extracellular traps and ferroptosis in diabetic wounds. Int Wound J 2023;20:3840–54. 37199077 10.1111/iwj.14231 PMC10588347

[6] Sass PA , Dąbrowski M, Charzyńska A, Sachadyn P. Transcriptomic responses to wounding: meta-analysis of gene expression microarray data. BMC Genomics 2017;18:850. 29115927 10.1186/s12864-017-4202-8 PMC5678747

[7] Pereira D , Sequeira I. A scarless healing tale: comparing homeostasis and wound healing of oral mucosa with skin and oesophagus. Front Cell Dev Biol 2021;9:682143. 34381771 10.3389/fcell.2021.682143 PMC8350526

[8] Zhou Z , Mei X, Hu K, Ma M, Zhang Y. Nanohybrid double network hydrogels based on a platinum nanozyme composite for antimicrobial and diabetic wound healing. ACS Appl Mater Interfaces 2023;15:17612–26. 37010097 10.1021/acsami.3c00459

[9] Zhu S , Yu Y, Ren Y, Xu L, Wang H, Ling X, Jin L, Hu Y, Zhang H, Miao C, Guo K. The emerging roles of neutrophil extracellular traps in wound healing. Cell Death Dis 2021;12:984. 34686654 10.1038/s41419-021-04294-3 PMC8536667

[10] Lu GB , Zhang CX, Li K, Gao K, Fu MQ, Lyu C, Quan ZX. Sinomenine ameliorates IL-1β-induced intervertebral disc degeneration in rats through suppressing inflammation and oxidative stress via Keap1/Nrf2/NF-κB signaling pathways. J Inflamm Res 2023;16:4777–91. 37881650 10.2147/JIR.S430423 PMC10596063

[11] Zhang SM , Ge GR, Qin Y, Li WH, Dong JL, Mei JW, Ma RX, Zhang XZ, Bai JX, Zhu C, Zhang WW, Geng DC. Recent advances in responsive hydrogels for diabetic wound healing. Mater Today Bio 2023;18:100508. 10.1016/j.mtbio.2022.100508 PMC9729074 36504542

[12] Liu H , Ding M, Wang H, Chen Y, Liu Y, Wei L, Cui X, Han Y, Zhang B, Zou T, Zhang Y, Li H, Chen R, Liu X, Cheng Y. Silver nanoparticles modified hFGF2-linking camelina oil bodies accelerate infected wound healing. Colloids Surf B Biointerfaces 2023;222:113089. 36527806 10.1016/j.colsurfb.2022.113089

[13] Fang Y , Xiu L, Xiao D, Zhang D, Wang M, Dong Y, Ye J. Sandwich-structured nanofiber dressings containing MgB2 and metformin hydrochloride with ROS scavenging and antibacterial properties for wound healing in diabetic infections. Adv Healthcare Mater n/a:2402452. 10.1002/adhm.202402452 39235573

[14] Chen J , Liu Y, Cheng G, Guo J, Du S, Qiu J, Wang C, Li C, Yang X, Chen T, Chen Z. Tailored hydrogel delivering niobium carbide boosts ROS-scavenging and antimicrobial activities for diabetic wound healing. Small 2022;18:e2201300. 35678523 10.1002/smll.202201300

[15] Chen HH , Guo YF, Zhang ZW, Mao WX, Shen CY, Xiong W, Yao YF, Zhao XZ, Hu YQ, Zou ZG, Wu JH. Symbiotic algae-bacteria dressing for producing hydrogen to accelerate diabetic wound healing. Nano Lett 2022;22:229–37. 34928162 10.1021/acs.nanolett.1c03693

[16] Parham S , Kharazi AZ, Bakhsheshi-Rad HR, Kharaziha M, Ismail AF, Sharif S, Razzaghi M, RamaKrishna S, Berto F. Antimicrobial synthetic and natural polymeric nanofibers as wound dressing: a review. Adv Eng Mater 2022;24:2101460.

[17] Parham S , Kharazi AZ, Bakhsheshi-Rad HR, Ghayour H, Ismail AF, Nur H, Berto F. Electrospun nano-fibers for biomedical and tissue engineering applications: a comprehensive review. Materials 2020;13:2153. 32384813 10.3390/ma13092153 PMC7254207

[18] Qu Z , Wang Y, Dong Y, Li X, Hao L, Sun L, Zhou L, Jiang R, Liu W. Intelligent electrospinning nanofibrous membranes for monitoring and promotion of wound healing. Mater Today Bio 2024;26:101093. 10.1016/j.mtbio.2024.101093 PMC11137601 38818528

[19] Gao Z , Wang Q, Yao Q, Zhang P. Application of electrospun nanofiber membrane in the treatment of diabetic wounds. Pharmaceutics 2021;14:6. 35056901 10.3390/pharmaceutics14010006 PMC8780153

[20] Liu Y , Li C, Feng Z, Han B, Yu D-G, Wang K. Advances in the preparation of nanofiber dressings by electrospinning for promoting diabetic wound healing. Biomolecules 2022;12:1727. 36551155 10.3390/biom12121727 PMC9775188

[21] Ji Y , Song W, Xu L, Yu D-G, Annie Bligh SW. A review on electrospun poly(amino acid) nanofibers and their applications of hemostasis and wound healing. Biomolecules 2022;12:794. 35740919 10.3390/biom12060794 PMC9221312

[22] Yang J , Xu L. Electrospun nanofiber membranes with various structures for wound dressing. Materials 2023;16:6021. 37687713 10.3390/ma16176021 PMC10488510

[23] Guzmán-Soria A , Moreno-Serna V, Canales DA, García-Herrera C, Zapata PA, Orihuela PA. Effect of electrospun PLGA/collagen scaffolds on cell adhesion, viability, and collagen release: potential applications in tissue engineering. Polymers (Basel) 2023;15:1079. 36904322 10.3390/polym15051079 PMC10006987

[24] Zhou X , Guo M, Wang Z, Wang Y, Zhang P. Rapid fabrication of biomimetic PLGA microsphere incorporated with natural porcine dermal aECM for bone regeneration. Regen Biomater 2024;11:rbae099. 39463918 10.1093/rb/rbae099 PMC11512121

[25] Martins C , Sousa F, Araújo F, Sarmento B. Functionalizing PLGA and PLGA derivatives for drug delivery and tissue regeneration applications. Adv Healthc Mater 2018;7:1701035. 10.1002/adhm.201701035 29171928

[26] Zhang X , Yu W, Zhang Y, Zhang W, Wang J, Gu M, Cheng S, Ren G, Zhao B, Yuan W-E. A hydrogen generator composed of poly (lactic-co-glycolic acid) nanofibre membrane loaded iron nanoparticles for infectious diabetic wound repair. J Colloid Interface Sci 2024;672:266–78. 38843679 10.1016/j.jcis.2024.05.222

[27] Liu Y , Xia B, Zhao R, Qin M, Weng X, Zeng Z, Deng K, Jiang H. Automatic in situ short-distance deposition of PLGA/PLLA composite nanofibrous membranes for personalized wound dressings. Nanoscale 2024;16:8546–62. 38596837 10.1039/d3nr06376c

[28] Guo J , Wei W, Zhao Y, Dai H. Iron oxide nanoparticles with photothermal performance and enhanced nanozyme activity for bacteria-infected wound therapy. Regen Biomater 2022;9:rbac041. 35812348 10.1093/rb/rbac041 PMC9258688

[29] Wang Y , Liu K, Wei W, Dai H. A multifunctional hydrogel with photothermal antibacterial and antioxidant activity for smart monitoring and promotion of diabetic wound healing. Adv Funct Materials 2024;34:2402531.

[30] Chen F , Luo Y, Liu X, Zheng Y, Han Y, Yang D, Wu S. 2D molybdenum sulfide-based materials for photo-excited antibacterial application. Adv Healthc Mater 2022;11:e2200360. 35385610 10.1002/adhm.202200360

[31] Zhang Y , Li X, Li D, Wei Q. A laccase based biosensor on AuNPs-MoS2 modified glassy carbon electrode for catechol detection. Colloids Surf B Biointerfaces 2020;186:110683. 31816461 10.1016/j.colsurfb.2019.110683

[32] Liu M , Zhu H, Wang Y, Sevencan C, Li BL. Functionalized MoS2-based nanomaterials for cancer phototherapy and other biomedical applications. ACS Mater Lett 2021;3:462–96.

[33] Wang Y , Liu K, Huang K, Wei W, Huang Y, Dai H. Photothermal antibacterial MoS2 composited chitosan hydrogel for infectious wound healing. Biomater Adv 2024;156:213701. 38039808 10.1016/j.bioadv.2023.213701

[34] Qiu L , Duan L, Lin H, Wang M, Liang H, Peng G, Yang X, Si Y, Yi S. Multifunctional and sprayable 2D MoS2/silk sericin bio-nanocomposite dressings with enhanced photothermal effect for infected wound healing. Adv Fiber Mater 2024;6:1074–91.

[35] Lu Y , Kang W, Yu Y, Liang L, Li J, Lu H, Shi P, He M, Wang Y, Li J, Chen X. Antibacterial and antioxidant bifunctional hydrogel based on hyaluronic acid complex MoS2–dithiothreitol nanozyme for treatment of infected wounds. Regen Biomater 2024;11:rbae025. 38605853 10.1093/rb/rbae025 PMC11009022

[36] Fu Y-G , Liu H-Q, Liu C, Lü Q-F. Ultralight porous carbon loaded co-doped MoS2 as an efficient electrocatalyst for hydrogen evolution reaction in acidic and alkaline media. J Alloys Compounds 2023;967:171748.

[37] Hong L , Li J, Liu F, Huang S, Zheng B, Ma X, Zhang Q, Zhao B, Yang C. Morphology-controllable fabrication of Ag@MoS2 composites with improved antioxidant activities at low Ag loading. Colloids Surf A Physicochem Eng Aspects 2020;596:124722.

[38] Zhu S , Lv Z, Jia X, Wang J, Li X, Dong M, Fan W. Pd nanoflakes epitaxially grown on defect MoS2 nanosheets for enhanced nitroarenes hydrogenation to anilines. Appl Catal B Environ Energy 2024;351:123958.

[39] Lu Y , Deng H, Pan T, Wang L, Zhang C, He H. Interaction between noble metals (Pt, Pd, Rh, Ir, Ag) and defect-enriched TiO_2_ and its application in toluene and propene catalytic oxidation. Appl Surf Sci 2022;606:154834.

[40] Jia Z , Yuan X, Wei J-a, Guo X, Gong Y, Li J, Zhou H, Zhang L, Liu J. A functionalized octahedral palladium nanozyme as a radical scavenger for ameliorating Alzheimer’s disease. ACS Appl Mater Interfaces 2021;13:49602–13. 34641674 10.1021/acsami.1c06687

[41] Chang M , Hou Z, Wang M, Yang C, Wang R, Li F, Liu D, Peng T, Li C, Lin J. Single-atom Pd nanozyme for ferroptosis-boosted mild-temperature photothermal therapy. Angew Chem Int Ed Engl 2021;60:12971–9. 33772996 10.1002/anie.202101924

[42] Chikukwa E , Meyer E, Mbese J, Zingwe N. Colloidal synthesis and characterization of molybdenum chalcogenide quantum dots using a two-source precursor pathway for photovoltaic applications. Molecules 2021;26:4191. 34299466 10.3390/molecules26144191 PMC8307795

[43] Kim Y , Kim J, Kim DH. Investigation on the enhanced catalytic activity of a Ni-promoted Pd/C catalyst for formic acid dehydrogenation: effects of preparation methods and Ni/Pd ratios. RSC Adv 2018;8:2441–8. 35541443 10.1039/c7ra13150j PMC9077440

[44] Barbosa R , Villarreal A, Rodriguez C, De Leon H, Gilkerson R, Lozano K. Aloe vera extract-based composite nanofibers for wound dressing applications. Mater Sci Eng C Mater Biol Appl 2021;124:112061. 33947555 10.1016/j.msec.2021.112061

[45] Zheng XD , Zhu YL, Sun YL, Jiao QJ. Hydrothermal synthesis of MoS2 with different morphology and its performance in thermal battery. J Power Sources 2018;395:318–27.

[46] Cai N , Li C, Han C, Luo X, Shen L, Xue Y, Yu F. Tailoring mechanical and antibacterial properties of chitosan/gelatin nanofiber membranes with Fe_3_O_4_ nanoparticles for potential wound dressing application. Appl Surface Sci 2016;369:492–500.

[47] Evranos B , Aycan D, Alemdar N. Production of ciprofloxacin loaded chitosan/gelatin/bone ash wound dressing with improved mechanical properties. Carbohydr Polym 2019;222:115007. 31320087 10.1016/j.carbpol.2019.115007

[48] D'Errico G , Vitiello G, De Tommaso G, Abdel-Gawad FK, Brundo MV, Ferrante M, De Maio A, Trocchia S, Bianchi AR, Ciarcia G, Guerriero G. Electron spin resonance (ESR) for the study of reactive oxygen species (ROS) on the isolated frog skin (*Pelophylax bergeri*): a non-invasive method for environmental monitoring. Environ Res 2018;165:11–8. 29655038 10.1016/j.envres.2018.03.044

[49] Ahmed W , Li S, Liang M, Kang Y, Liu X, Gao C. Multifunctional drug- and AuNRs-loaded ROS-responsive selenium-containing polyurethane nanofibers for smart wound healing. ACS Biomater Sci Eng 2024;10:3946–57. 38701357 10.1021/acsbiomaterials.4c00363

[50] Liu J , Xie X, Wang T, Chen H, Fu Y, Cheng X, Wu J, Li G, Liu C, Liimatainen H, Zheng Z, Wang X, Kaplan DL. Promotion of wound healing using nanoporous silk fibroin sponges. ACS Appl Mater Interfaces 2023;15:12696–707. 36855948 10.1021/acsami.2c20274

[51] Orive G , Carcaboso AM, Hernández RM, Gascón AR, Pedraz JL. Biocompatibility evaluation of different alginates and alginate-based microcapsules. Biomacromolecules 2005;6:927–31. 15762661 10.1021/bm049380x

[52] Yang LY , Jiang ZY, Zhou LH, Zhao KL, Ma X, Cheng GS. Hydrophilic cell-derived extracellular matrix as a niche to promote adhesion and differentiation of neural progenitor cells. RSC Adv 2017;7:45587–94.

[53] Huang W , Zhang YX, Zhang Y, Fang DQ, Schauer JJ. Optimization of the measurement of particle-bound reactive oxygen species with 2′,7′-dichlorofluorescin (DCFH). Water Air Soil Pollution 2016;227:164.

[54] Cui Y , Xu N, Xu W, Xu GX. Mesenchymal stem cells attenuate hydrogen peroxide-induced oxidative stress and enhance neuroprotective effects in retinal ganglion cells. In Vitro Cell Dev Biol Anim 2017;53:328–35. 27864663 10.1007/s11626-016-0115-0

[55] Khan AA , Ullah S, Amin R. Correction to: optimal control analysis of COVID-19 vaccine epidemic model: a case study. Eur Phys J Plus 2022;137:198. 35136704 10.1140/epjp/s13360-022-02420-4 PMC8814565

[56] Ding LH , Ren F, Liu Z, Jiang ZL, Yun BF, Sun Q, Li Z. Size-dependent photothermal conversion and photoluminescence of theranostic NaNdF4 nanoparticles under excitation of different-wavelength lasers. Bioconjug Chem 2020;31:340–51. 31751118 10.1021/acs.bioconjchem.9b00700

[57] Chiang ELC , Lee S, Medriano CA, Li LY, Bae S. Assessment of physiological responses of bacteria to chlorine and UV disinfection using a plate count method, flow cytometry and viability PCR. J Appl Microbiol 2022;132:1788–801. 34637587 10.1111/jam.15325

