# Supplementary material for: A multifunctional photothermal electrospun PLGA/MoS2@Pd nanofiber membrane for diabetic wound healing
Source: Regen Biomater. 2024 Dec 14;12:rbae143. doi: 10.1093/rb/rbae143 (PMC11754638; doi:10.1093/rb/rbae143)
Supplement: rbae143_Supplementary_Data [file rbae143_supplementary_data.docx]

**A multifunctional photothermal electrospun PLGA/MoS_2_@Pd nanofiber membrane for diabetic wound healing**

Zhengrong Chen ^1, 2, #^, Quansheng Mo ^3, #^, Dandan Mo ^1, #^, Xiaomin Pei ^1, 4^, Anru Liang ^5^, Jinhong Cai ^1^, Bo Zhou ^1^, Li Zheng ^1, 4,^ *, Hongmian Li ^6,^ *, Feiying Yin ^1, 4,^ *, Jinmin Zhao ^1, 7^

^1^ Guangxi Engineering Center in Biomedical Material for Tissue and Organ Regeneration, Collaborative Innovation Centre of Regenerative Medicine and Medical BioResource Development and Application Co-constructed By the Province and Ministry, Guangxi Key Laboratory of Regenerative Medicine, The First Affiliated Hospital of Guangxi Medical University, Nanning, Guangxi 530021, China.

^2^ National & Regional United Engineering Lab of Tissue Engineering, Department of Orthopedics, Southwest Hospital, Third Military Medical University (Army Medical University), Chongqing, 400038, China.

^3^ Department of Traditional Chinese Medicine, The Ninth People's Hospital of Nanning, Binyang, 530409, Guangxi, China.

^4^ Life Sciences Institute, Guangxi Medical University, Nanning, Guangxi, 530021, China.

^5^ Department of burns and Plastic Surgery, the Third Affiliated Hospital of Guangxi Medical University & the Second Nanning People's Hospital, Nanning, 530031, China

^6^ Department of Plastic and Reconstructive Surgery, The People’s Hospital of Guangxi Zhuang Autonomous Region & Research Center of Medical Sciences, Guangxi Academy of Medical Sciences, Nanning, 530021, China.

^7^ Department of Orthopedics, The First Affiliated Hospital of Guangxi Medical University, Nanning, 530021, China.

* Corresponding authors.

Email addresses: zhengli224@163.com (L. Zheng), lihongmian@gxmu.edu.cn (H. Li) and yinfeiying@yeah.net (F. Yin).

^#^ The authors contributed equally to this work.


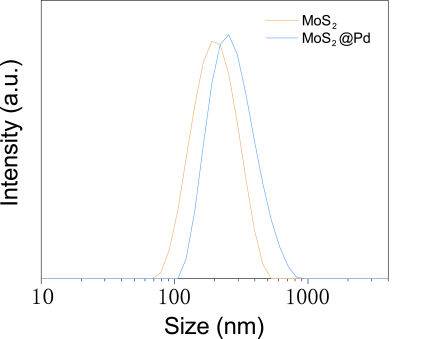


**Figure S1.** Size distribution of MoS_2_ and MoS_2_@Pd.


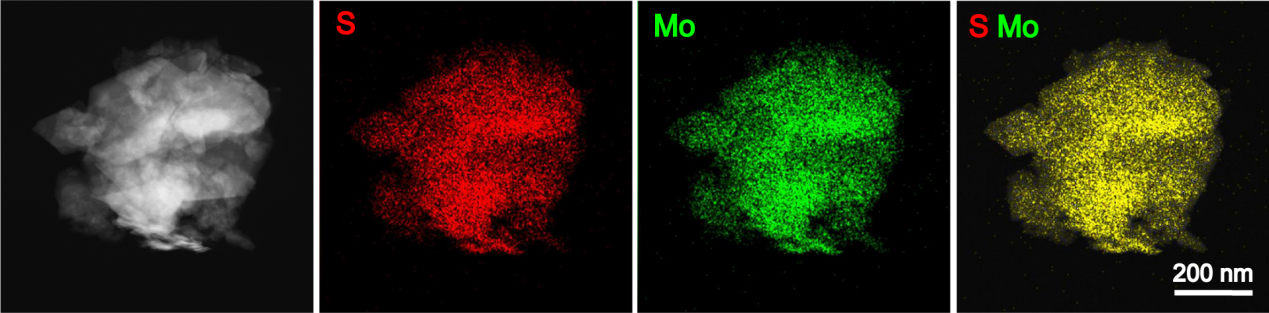


**Figure S2.** Corresponding elemental mappings of S, Mo in MoS_2_.


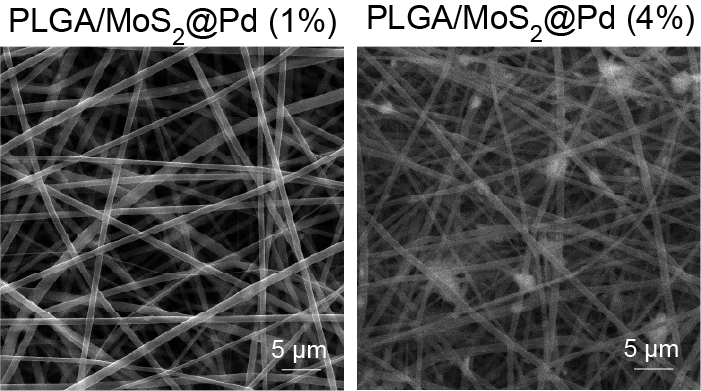


**Figure S3.** SEM images of PLGA/MoS_2_@Pd with 1% and 4% MoS_2_@Pd loading.


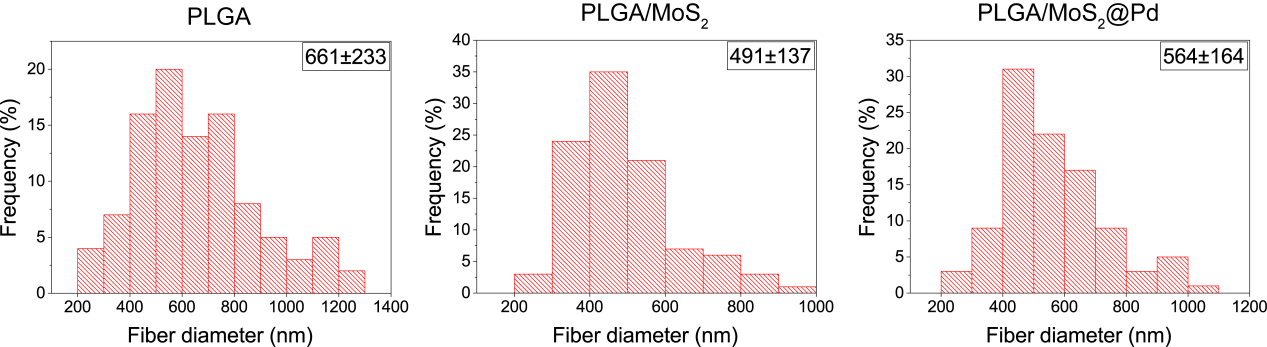


**Figure S4.** Diameter distribution of PLGA, PLGA/MoS_2_, and PLGA/MoS_2_@Pd nanofibers.


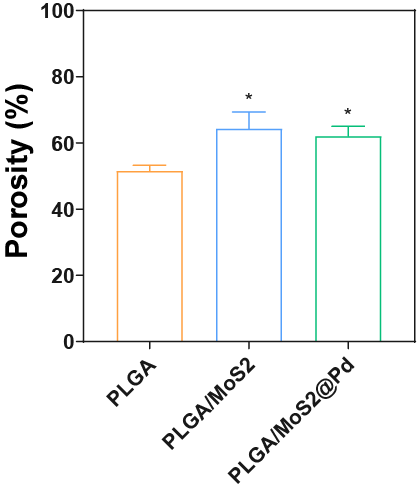


**Figure S5.** Porosity of PLGA, PLGA/MoS_2_, and PLGA/MoS_2_@Pd nanofiber membranes.


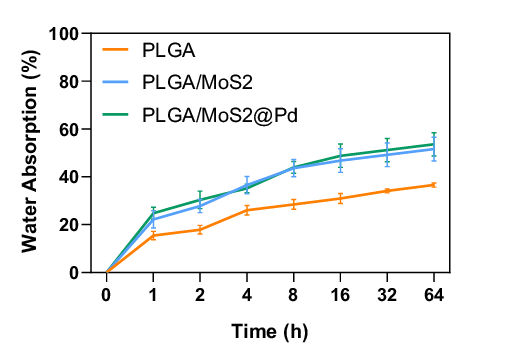


**Figure S6.** Water absorption capacity of PLGA, PLGA/MoS_2_, and PLGA/MoS_2_@Pd nanofiber membranes.


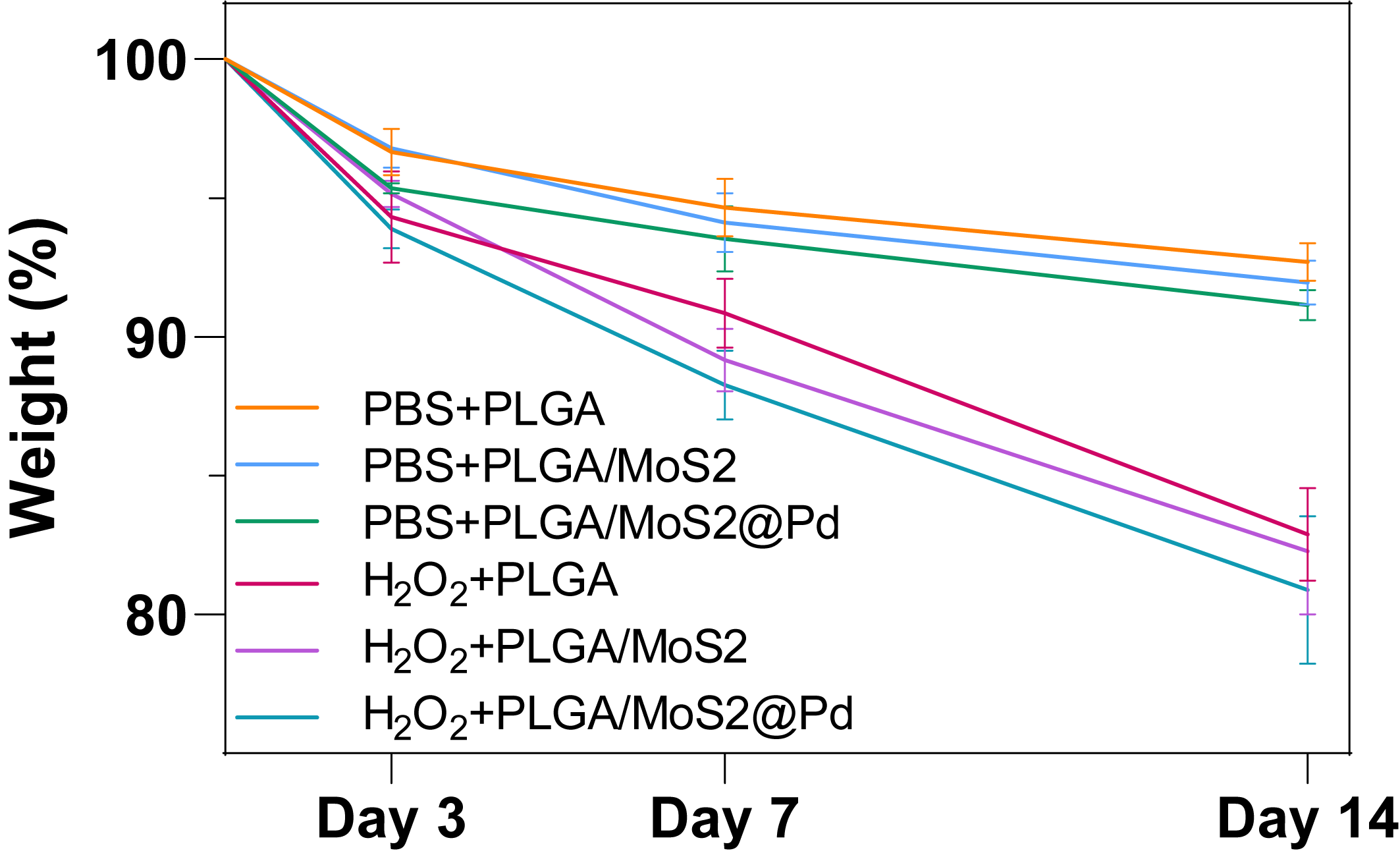


**Figure S7.** Degradation rates of PLGA, PLGA/MoS_2_, and PLGA/MoS_2_@Pd nanofiber membranes in PBS and H_2_O_2_ solution.


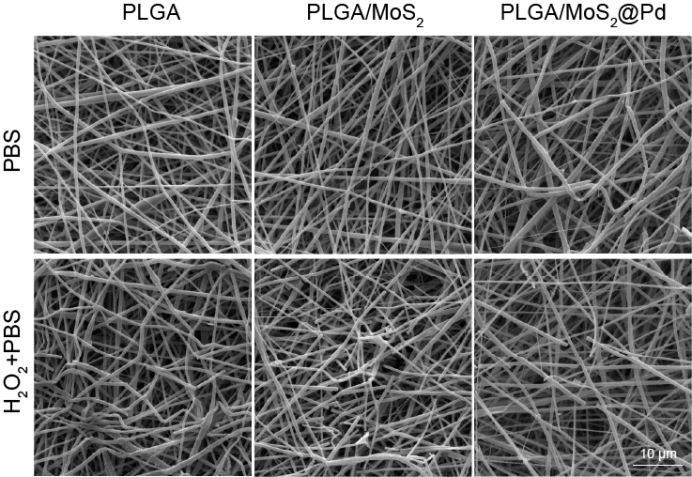


**Figure S8.** SEM images of PLGA, PLGA/MoS_2_, and PLGA/MoS_2_@Pd nanofiber membranes after 14 days of treatment in PBS and H_2_O_2_ solution.


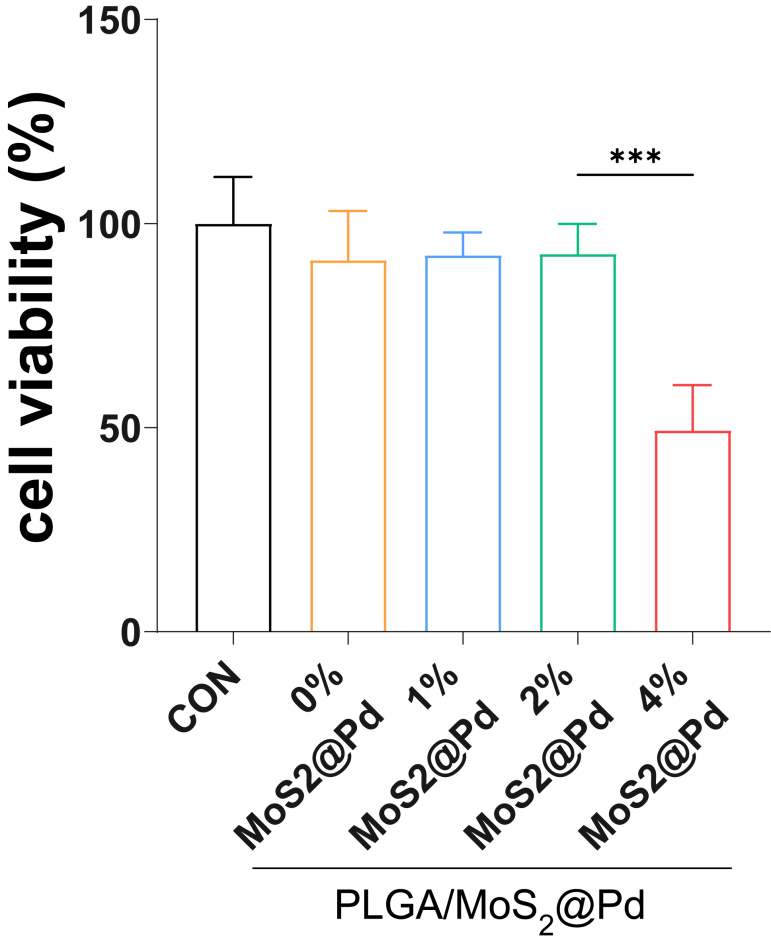


**Figure S9.** Relative cell viability of PLGA/MoS_2_@Pd with different MoS_2_@Pd loading.


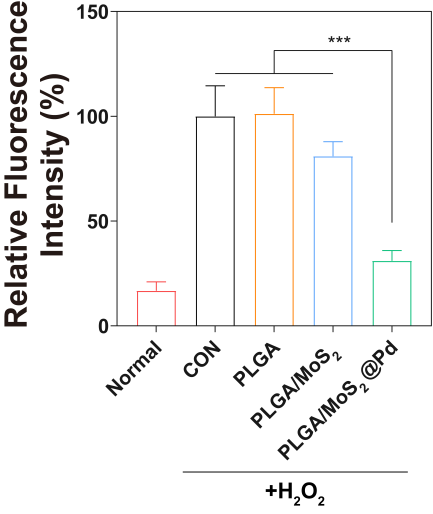


**Figure S10.** Quantified results of fluorescent intensity of intracellular ROS levels.


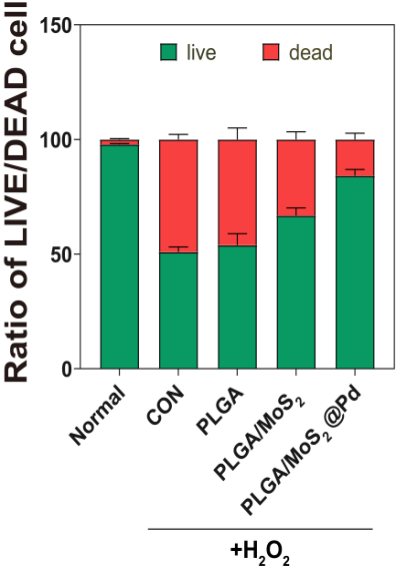


**Figure S11.** Quantified results of live/dead staining of H_2_O_2_-induced fibroblasts.


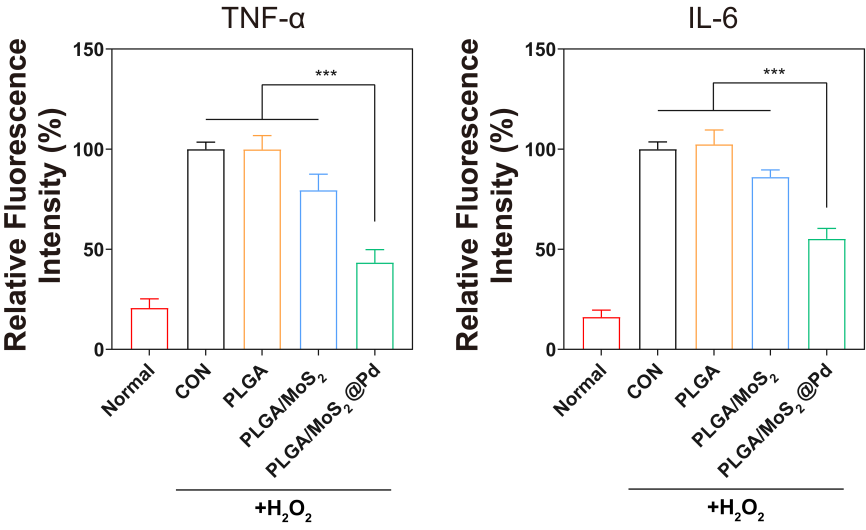


**Figure S12.** Quantified results of fluorescence intensity of inflammatory factors (TNF-α and IL-6).


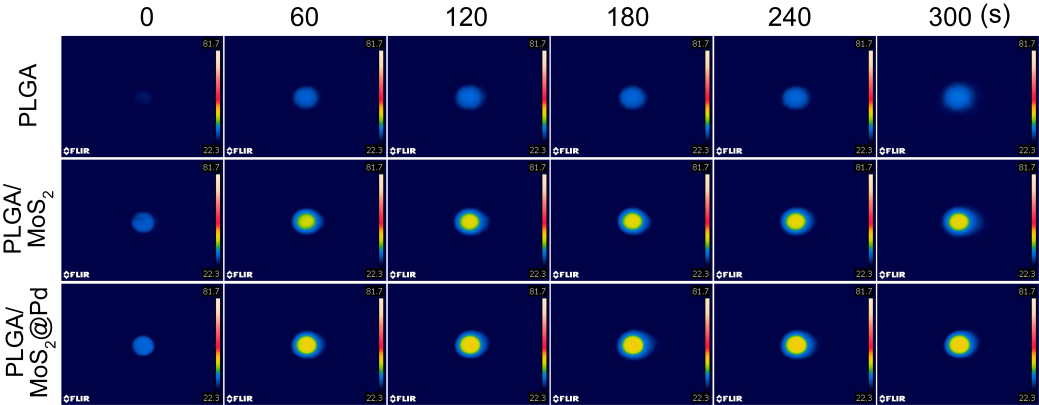


**Figure S13.** Temperature change images of PLGA, PLGA/MoS_2_, and PLGA/MoS_2_@Pd at different time points (0, 60, 120, 180, 240 and 300 s) under NIR irradiation (808 nm, 0.5 W/cm^2^).


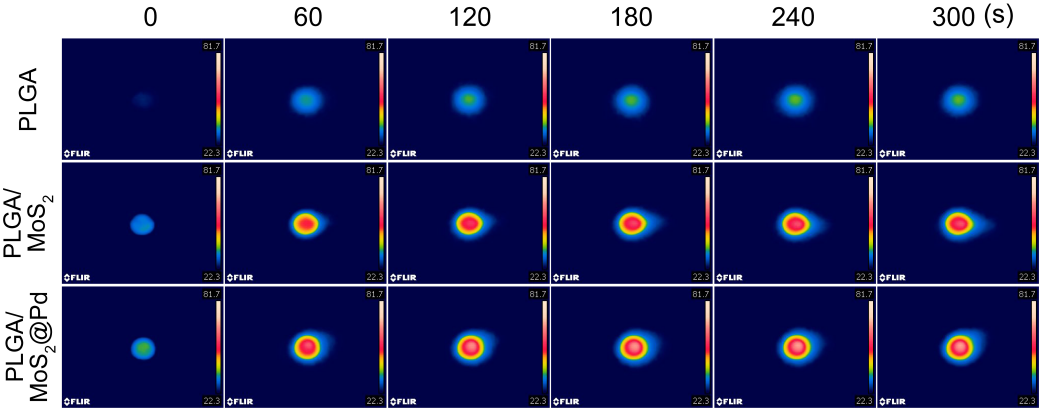


**Figure S14.** Temperature change images of PLGA, PLGA/MoS_2_, and PLGA/MoS_2_@Pd at different time points (0, 60, 120, 180, 240 and 300 s) under NIR irradiation (808 nm, 1.5 W/cm^2^).


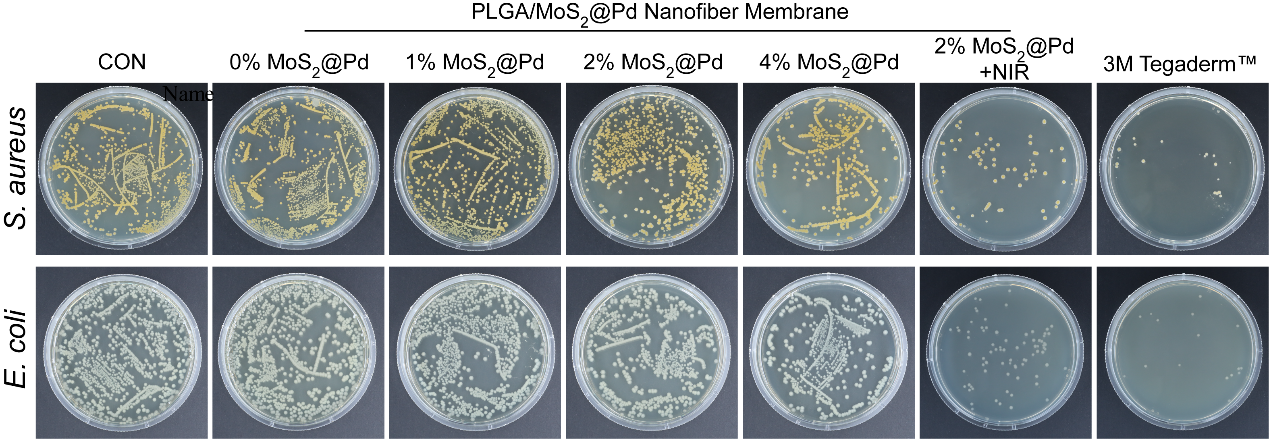


**Figure S15.** Antibacterial effects of various treatments on *S. aureus* and *E. coli*.


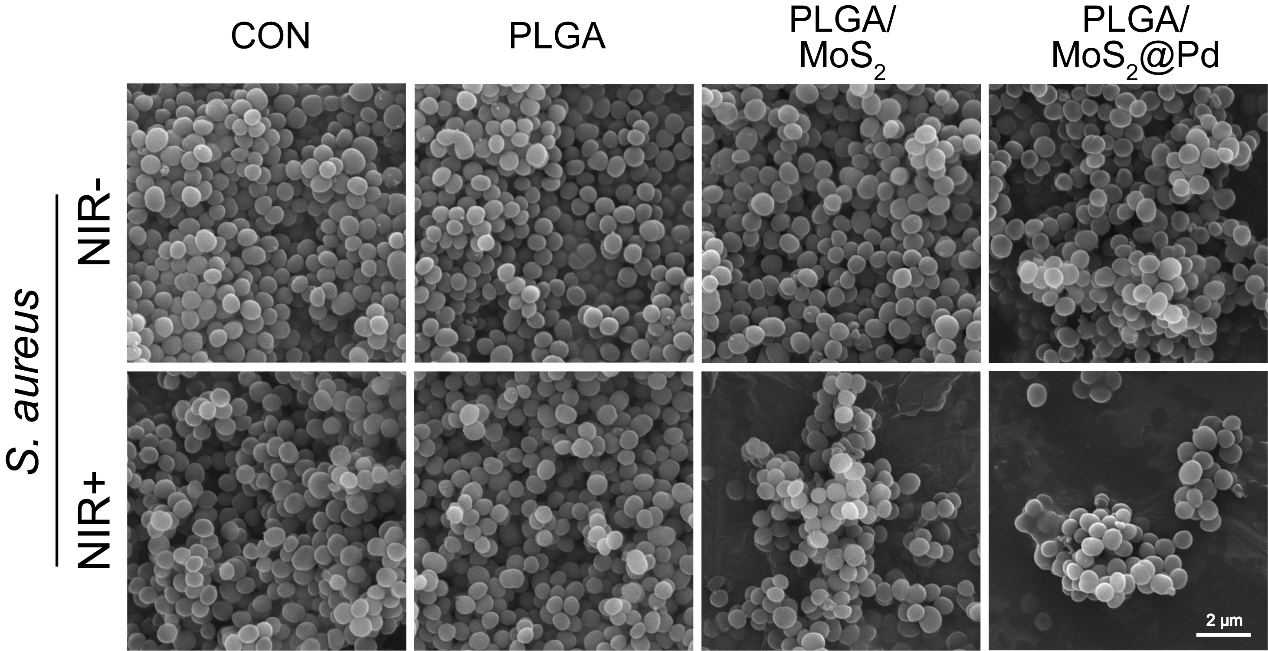


**Figure S16.** SEM morphologies of *S. aureus* after different treatments.


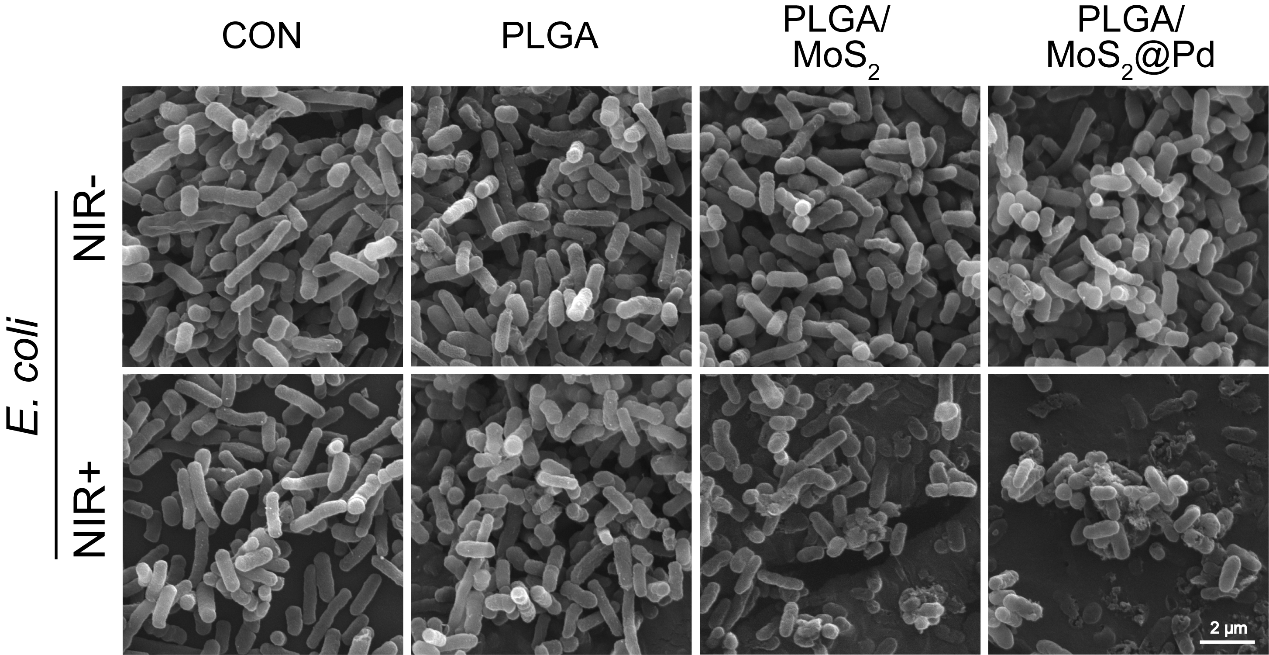


**Figure S17.** SEM morphologies of *E. coli* after different treatments.


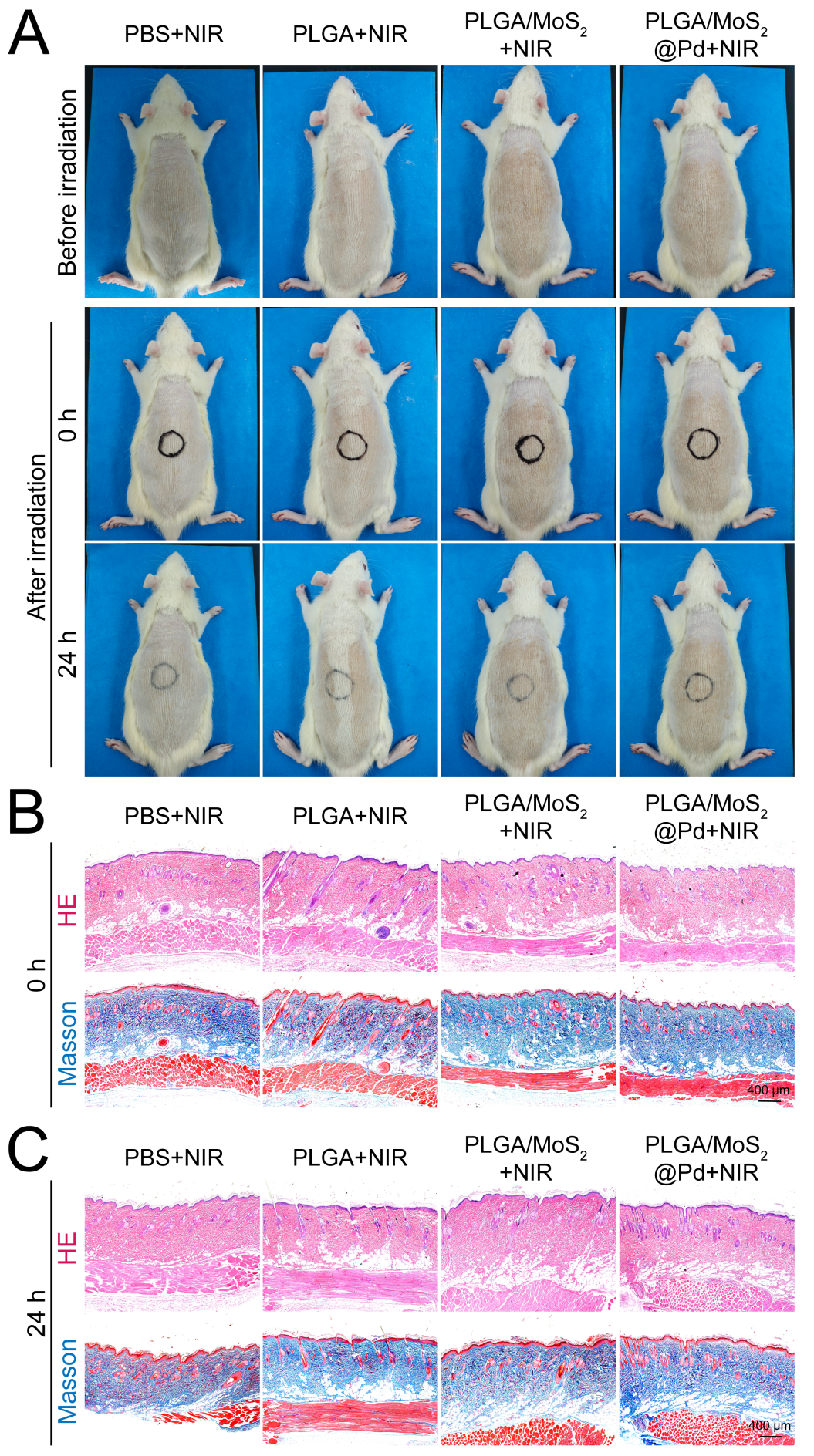


**Figure S18.** The verification results of temperature safety generated by NIR irradiation. (A) Macroscopic images of skin of each group with different treatments before and after NIR irradiation. The results of H&E and Masson staining of skin tissue removed from SD rats at (B) 0 h and at (C) 24 h.


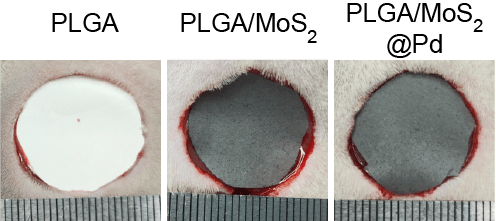


**Figure S19.** Macroscopic images of wound models.


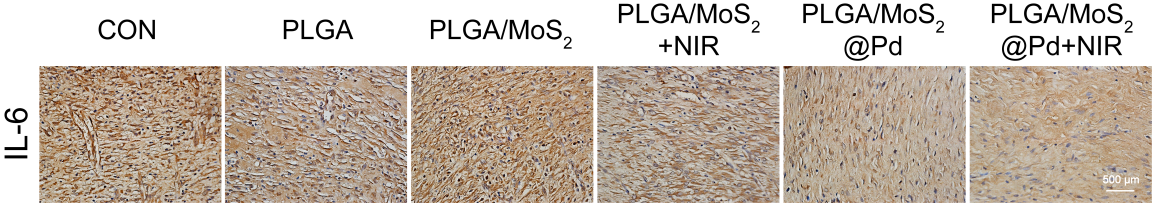


**Figure S20.** Immunohistochemistry images for IL-6 after different treatments on day 14 post-operation.


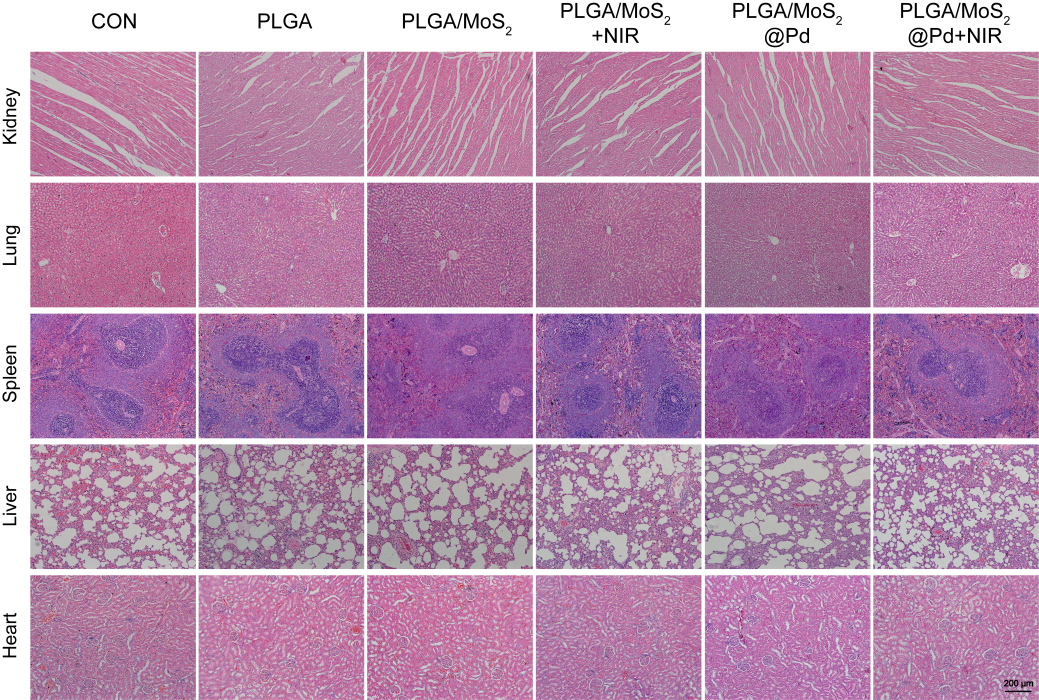


**Figure S21.** H&E staining of heart, liver, spleen, lung and kidney after different treatments on day 14 post-operation.

**Table S1.** Primers in qRT-PCR analysis.

| Gene Forward primer (5'--3') Reverse primer (5'--3') | | |
| --- | --- | --- |
| *TNF-α* | 5'-GGAGGGAGAACAGCAACTCC -3' | 5'- TCTGCCAGTTCCACATCTCG-3' |
| *IL-1β* | 5'-TGCCACCTTTTGACAGTGATG-3' | 5'-ATGTGCTGCTGCGAGATTTG-3' |
| *IL-6* | 5'-GAGAAATCGATGACAGCGCC-3' | 5'- GATGAATTGGATGGTCTTGGTCC -3' |
| *GAPDH* | 5'-ACTTGAAGGGTGGAGCCAAA-3' | 5'-GCCCTTCCACAATGCCAAAG-3' |

**Table S2.** The ratio of S, Mo, and Pd of MoS_2_ and MoS_2_@Pd NPs by EDX.

| Name | S% (±SD) | Mo% (±SD) | Pd% (±SD) |
| --- | --- | --- | --- |
| MoS_2_ | 57.92 ± 2.08 | 42.08 ± 0.78 | / |
| MoS_2_@Pd | 55.02 ± 1.98 | 39.64 ± 1.09 | 5.34 ± 1.71 |

**Table S3.** Nanofiber membranes with different ratios.

| Samples | PLGA  (mg/mL) | Nanoparticles  (mg/mL) |
| --- | --- | --- |
| PLGA (0%) | 100 | 0 |
| PLGA/MoS_2_@Pd (1%) | 100 | 1 MoS_2_@Pd |
| PLGA/MoS_2_@Pd (2%) | 100 | 2 MoS_2_@Pd |
| PLGA/MoS_2_@Pd (4%) | 100 | 4 MoS_2_@Pd |
| PLGA/MoS_2_ (2%) | 100 | 2 MoS_2_ |
